# Naturally based ionic liquids with indole-3-acetate anions and cations derived from cinchona alkaloids†

**DOI:** 10.1039/d1ra04805h

**Published:** 2021-08-13

**Authors:** Tomasz Rzemieniecki, Tomasz Kleiber, Juliusz Pernak

**Affiliations:** Department of Chemical Technology, Poznan University of Technology Berdychowo 4 Poznan 60-965 Poland juliusz.pernak@put.poznan.pl; Department of Agronomy, Horticulture and Bioengineering, Poznan University of Life Sciences Zgorzelecka 4 Poznan 60-198 Poland

## Abstract

The use of highly efficient methods and natural raw materials in syntheses of new biologically active substances addresses the current challenges in this area: ensuring the highest possible efficacy at low concentrations and reducing negative environmental impact. In the present study, we applied this strategy to obtain a new group of ionic liquids containing the indole-3-acetate anion, which is a well-known plant growth hormone, and a cation derived from a cinchona alkaloid – quinine or quinidine. A comparison of the derivatization kinetics of both alkaloids was also carried out, and the use of a quaternary quinidine derivative as a source of biologically active ionic liquids is described here for the first time. The structures of the obtained compounds were fully confirmed based on spectral methods. According to analyses of the effects of the obtained compounds on the growth and development of lettuce plants (*Lactuca sativa* L.), the ionic liquids obtained with indole-3-acetate anions exhibited activity at a concentration of 0.5 mg dm^−3^, and the length of the alkyl substituent in the alkaloid-derived cation or the chirality of this cation is crucial in determining the biological activity of the compound. In the cases of several salts containing the 1-alkylquininium cation, we recorded significant, beneficial changes in micronutrient content, which directly translated into plant nutritional value, while no signs of phytotoxicity were observed. Analyses of relevant physicochemical properties (*e.g.*, with differential scanning calorimetry, thermogravimetric analysis and solubility analysis) as well as microbial toxicity tests were also performed to evaluate the environmental impacts of the products. The promising results of our study indicate significant potential for application of these new ionic liquids derived from cinchona alkaloids.

## Abstract 2

The transformation of a plant hormone into an alkaloid-based ionic liquid results in unique changes in physicochemical and biological properties.

## Graphical abstract

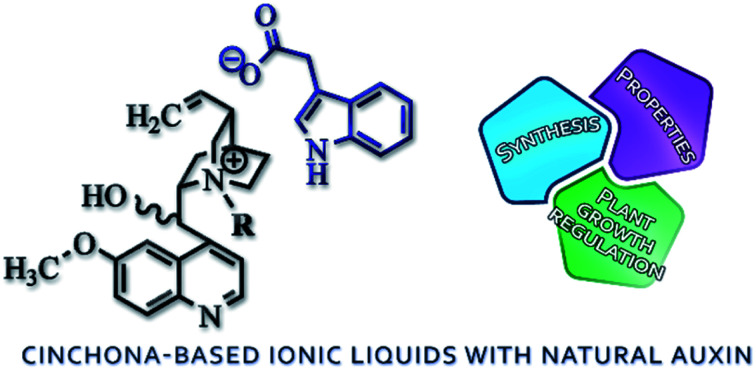

## Introduction

One of the most important challenges in the sustainable development of modern biologically active chemicals is the design of novel ingredients characterized by unique modes of action.^1–5^ It should be stressed that it is possible to obtain products with a very high level of biocompatibility when naturally derived compounds with particularly strong biological effects on other living organisms are used as substrates.^6–8^ Alkaloids are an interesting group of potential models for new biologically active compounds; this is a vast group of natural, organic substances with high structural diversity, whose common feature is the presence of a heterocyclic group containing a nitrogen atom with basic character.^9,10^ Compounds in this group are characterized by the use of small doses with significant activity towards a broad spectrum of living organisms, which determines their use as beneficial active substances with unique types of effectiveness, *e.g.*, cytotoxic activity of camptothecin derivatives towards malignant tumours, including those from small cell lung cancer,^8^ as well as indole alkaloids and their derivatives that serve as antidepressant agents.^11^

The strategy of using highly biologically active ingredients can be successfully applied in modern agrotechnology. For example, the use of compounds from a group of plant growth regulators, including auxins, gibberellins or cytokinins,^12,13^ is widespread and results in the desired quantity and quality of yields at the right time. Auxins are a particularly attractive group of growth regulators, of which indole-3-acetic acid (IAA) is the best-known example. Their high application potential is due to the fact that they exhibit biological activity at very low concentrations (as low as 10^−12^ mol dm^−3^)^14^ and, at the same time, beneficially affect a number of important crop plant factors, including shoot growth,^15^ rooting,^16,17^ nutrient uptake and plant nutritive value,^18^ as well as responses to stress factors.^19^ The undoubted advantages of IAA also include its biocompatibility and biodegradability^20,21^ and the fact that it shows minor toxic effects for mammals (LD_50_ > 1000 mg kg^−1^; rat, oral).^22^

According to literature reports from the last decade, conversion of conventional solid active ingredients into liquid forms allows for a number of advantages; these include higher bioavailability^23^ and the absence of spontaneous crystallisation from the working solution.^24,25^ In practice, this effect can be easily achieved by converting the nonionic form of the active ingredient into an ionic liquid (IL).^5,25^ In the course of research on third generation ILs (*i.e.*, ILs designed for targeted biological activity),^26,27^ compounds characterised by specific effects on plants, including herbicidal activity,^28^ stimulated resistance against phytoviruses^29,30^ or limited plant growth,^31^ have been discovered. However, converting compounds from the auxin group into biologically active ILs constitutes a very recent approach. In 2020, an article was published describing the synthesis and characterization of ILs containing choline cation and its alkyl derivatives with the indole-3-butyrate anion; the latter is a protoauxin, and it is converted into IAA in plant tissues.^32^ In addition, it should be noted that protoauxin and its ionic derivatives showed activity at concentrations as low as 0.5 mg dm^−3^ per dose of indole-3-butyrate anion. To the best of our knowledge, no studies regarding the influence of IAA-based ILs on plants have been published to date.

In framing our study, we attempted to combine both concepts described above by synthesising ILs comprising ions originating from highly biologically active substances in low concentrations: indole-3-acetic acid anions and cations derived from alkaloids. Two cinchona alkaloids, which are mutual pseudoenantiomers, quinine and quinidine, were chosen as sources of cations. Only a few groups of ILs with quinine-derived cations have been described thus far,^33–35^ while there are no literature reports on the preparation of ILs with a quinidine-based cation. According to basic assumptions concerning 3rd generation ILs, the novel forms of IAA designed herein should be characterised by more favourable physicochemical properties compared to the acidic form of IAA. Moreover, it has been confirmed that cations derived from cinchona alkaloids can result in unique changes in the biological activity of the new ILs,^35^ which should translate directly into a significant increase in the potential for application of the active substances.

## Materials and methods

### Materials

Quinine (purity >98%), quinidine (purity >99%) bromoethane (purity 98%), 1-bromobutane (purity 99%), 1-bromohexane (purity 98%), 1-bromooctane (purity 98%), 1-bromodecane (purity 98%) 1-bromododecane (purity 97%), indole-3-acetic acid (purity >97%), all solvents (methanol, ethanol, dimethyl sulfoxide, acetonitrile, acetone, 2-propanol, ethyl acetate, diethyl ether, chloroform, toluene, hexane) and ion-exchange resin AmberTec™ UP550 OH were purchased from Merck KGaA, (Darmstadt, Germany). Concentrated acids used in chemical composition analyses: sulfuric acid (>95%), nitric acid (ultrapure) and perchloric acid (analytically pure) were supplied by Avantor Performace Materials SA (Gliwice, Poland). Indole-3-acetic acid was additionally purified by double recrystallisation from boiling water. No other reagents or solvents were subjected to purification before use. Deionized water for solubility measurements with a conductivity <0.1 μS cm^−1^ was obtained from demineralizer HLP Smart 1000 (Hydrolab, Straszyn, Poland).

### Synthetic procedures

#### Synthesis of quaternary ammonium bromides

1-Alkylquininium bromides containing substituents ranging from ethyl to dodecyl were synthesized according to a previously disclosed methodology.^35^ 1-Ethylquinidinium bromide was synthesized *via* the following method: 0.015 mol of quinidine was placed in a 50 cm^3^ EasyMax™ reactor equipped with a stir bar, a condenser, a temperature sensor, and a 9.5 mm AgX probe with a diamond tip connected with a ReactIR™ 15 (Mettler Toledo) FT-IR spectrometer. Then, the reagent was dissolved in 20 cm^3^ of DMSO, and a stoichiometric amount of bromoethane was introduced into the reaction system through a dropping funnel. The reaction was conducted for 48 h at 35 °C. The postreaction mixture was transferred to a dropping funnel and introduced dropwise into a vigorously stirred system of 2-propanol and diethyl ether (1 : 20, v/v). After 60 min of continuous stirring, the precipitate was filtered, dried and purified by double recrystallisation from boiling 2-propanol. Finally, the obtained crystalline solid was dried under reduced pressure at 50 °C for 18 h.

#### Synthesis of indole-3-acetate cinchona derivatives

All 1-alkylquininium indole-3-acetates and 1-ethylquinidinium indole-3-acetate were synthesized according to a previously disclosed methodology.^35^ The respective 1-alkylquininium bromide or 1-ethylquinidinium bromide (0.005 mol) was dissolved in ethanol (20 cm^3^) and introduced into a 50 cm^3^ EasyMax™ reactor. In the next step, 10 cm^3^ of the anionic resin AmberTec™ UP550 OH was added in the form of an ethanolic suspension, and the system was stirred for 30 min at 25 °C. The resin was filtered off and washed three times with small volumes (5 cm^3^) of ethanol. The obtained solutions of quaternary hydroxides were subsequently neutralized using stoichiometric amounts of indole-3-acetic acid, which was added through a dropping funnel in the form of ethanolic solution. Next, the solvent and water were removed using a rotary evaporator. The obtained products were thoroughly dried under reduced pressure (5 mbar) at 50 °C for 18 h. All synthesized ILs were stored under reduced pressure over P_4_O_10_. In addition, a binary mixture of quinine and indole-3-acetic acid was obtained *via* one-step homogenization in ethanol. The system was vigorously stirred for 30 min, and in the next step, the solvent was evaporated.

### Characterisation of compounds

#### Spectral analysis

The ^1^H NMR spectra were acquired using VNMR-S 400 MHz (Varian Inc. USA) spectrometer operating at the frequency of 400 MHz. TMS was used as the internal standard. The ^13^C NMR spectra were obtained using the same apparatus operating at 100 MHz. The FT-IR spectra were recorded on IFS 66v/S spectrometer (Bruker Optics, Ettlinger, Germany). The data were sampled from 4000 to 400 cm^−1^ and visualized using Spectragryph 1.2.13 (ref. 36) software.

#### Thermal analysis

Thermal transition temperatures were determined by differential scanning calorimetry, using a Mettler Toledo Star^e^ DSC1 (Mettler Toledo, Leicester, UK) unit, under nitrogen. Samples between 4.5 and 7.0 mg were placed in aluminum pans and heated from 25 to 120 °C at a heating rate of 10 °C min^−1^. Then, the samples were cooled with an intracooler at a cooling rate of 10 °C min^−1^ to 100 °C and then heated again to 120 °C at the same heating rate. Thermal gravimetric analysis was performed using a Mettler Toledo Star^e^ TGA/DSC1 unit (Mettler Toledo, Leicester, UK) under nitrogen. Samples between 4.0 and 5.0 mg were placed in aluminum pans and heated from 30 to 450 °C at a heating rate of 10 °C min^−1^.

#### Solubility

Water and 9 organic solvents with a range of polarities were selected for solubility tests. The solvents were arranged in order of decreasing polarity according to the Snyder polarity index: water, 9.0; methanol, 6.6; dimethylsulfoxide (DMSO), 6.5; acetonitrile, 6.2; acetone, 5.4; 2-propanol, 4.3; ethyl acetate, 4.3; chloroform, 4.1; toluene, 2.3 and hexane, 0.0. The solubility measurements were performed according to the methodology described in Vogel's Textbook of Practical Organic Chemistry.^37^ The sample of an analysed IL (0.1 ± 0.0001 g) was introduced into a specific volume of solvent. The measurements were conducted at 25 °C. Depending on the volume of solvent used, three types of behaviour were recorded: “ready solubility” applies to ILs that dissolved in 1 cm^3^ of the solvent (>10.0% m/v), “limited solubility” applies to compounds that dissolved in 3 cm^3^ of the solvent (between 3.33–10.0% m/v), and “low solubility” applies to ILs that did not dissolve in 3 cm^3^ of the solvent (<3.33% m/v).

### Biological activity

#### Plant cultivation

Biological studies were conducted in a controlled growth chamber. The aim was to evaluate the effects of ILs with indole-3-acetate anions on the chemical composition, yield and photosynthetic activity (measured as chlorophyll fluorescence) of lettuce (*Lactuca sativa* L. ‘Zeralda’); this was used as a model plant and was grown with hydroponic cultivation. During the tests (carried out from November 2019 to January 2020), the ILs were applied in the nutrient solution at a concentration of 0.5 mg dm^−3^, which was assumed to be a favourable value during previous studies. Indole-3-acetic acid at the same concentration was used as a reference substance.

The experiment was established using the systematic design in 6 replications. During the experiment, the following stable conditions were maintained: photoperiod of 14/10 h; temperature of 16.5 °C ± 0.5 °C; and relative humidity (RH) 70–80%. The photosynthetic photon flux density (PPFD) was 235 to 250 μmol m^−2^ s^−1^; this was supplied by LED lamps that provided peak red wavelengths (approximately 660 nm) and blue wavelengths (approximately 455 nm). The quantum flux density was measured with the SunScan Canopy Analysis System (SS1, Delta-T Devices Ltd., Cambridge, UK). The seeds were sown individually in rockwool.

The rockwool was soaked in standard nutrient solution 48 h before experiments. The germination of seeds was conducted in a controlled growth chamber. Seedlings (in the 4–5 leaf phase) were placed in nutrient solution on rockwool blocks (Grodan, 100 × 100 × 65 mm). The plants were subsequently placed in a growth container forming a hydroponic stagnant system; nutrient solutions were dosed according to plant requirements. Application of the tested compounds was started according to the start of plant cultivation in the hydroponic system. The standard nutrient solution for plant fertigation contained (mg dm^−3^): N–NH_4_ < 10, N–NO_3_ 150, P–PO_4_ 50, K 150, Ca 150, Mg 50, Fe 3.00, Mn 0.5, Zn 0.44, Cu 0.03, B 0.011; pH 5.50, and EC 1.8 mS cm^−1^. The following fertilizers were used to prepare nutrient solution: potassium nitrate (13% N–NO_3_, 38.2% K), calcium nitrate (14.7% N–NO_3_, 18.5% Ca), monopotassium phosphate (22.3% P, 28.2% K), potassium sulfate (44.8% K, 17% S), magnesium sulfate (9.9% Mg, 13% S), Librel FeDP7 (7% Fe), manganese sulfate (32.3% Mn), copper sulfate (25.6% Cu), borax (11.3% B) and sodium molybdate (39.6% Mo). Nitric acid (38%) was used to regulate the pH.

At the end of the experiment, the following parameters were determined according to procedures described previously;^38^ the weight of the lettuce head (g), dry matter content (%) and relative water content (%).
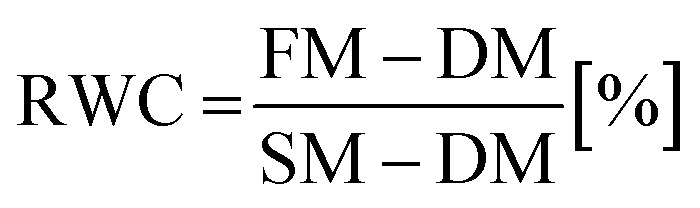
FM – leaf fresh mass at the time of collection, SM – leaf mass after 24 h of saturation in distilled water, DM – leaf dry mass.

#### Chemical composition analyses

Chemical composition analyses were performed for the aboveground parts of the plants. The accuracy of the methods of chemical analyses and the precision of analytical measurements of nutrients were previously tested in the laboratory by means of analysis of the reference material for strawberry leaves (LCG7162), which was certified by the IRMM (Institute for Reference Materials and Measurements) in Belgium. Samples were dried for 48 h at 45–50 °C and then finely ground. Prior to mineralization, the plant material was additionally dried for 1 h at 105 °C. The contents of N, P, K, Ca, Mg and Na were assessed after plant mineralization in concentrated sulfuric acid. For analyses of total Fe, Mn, Zn and Cu, the plant material was digested in a mixture of ultrapure concentrated nitric and perchloric acids in a 3 : 1 ratio. After mineralization, the following determinations were performed: N-total using the distillation method according to Kjeldahl in a Parnas Wagner apparatus; P, which was colourimetrically determined with ammonium molybdate; and K, Ca, Mg, Na, Fe, Mn, Zn, and Cu using flame atomic absorption spectroscopy (FAAS, Carl Zeiss Jena 5, Germany).

#### Physiological measurements

At the end of the experiment, the following parameters were measured: *F*_0_ (initial fluorescence), *F*_M_ (maximal fluorescence intensity), *F*_V_ (maximal variable fluorescence), *F*_V_/*F*_M_ (maximum photochemical quantum PSII after dark adaptation), *F*_V_/*F*_0_ (potential activity of PSII), PI_ABS_ (photosynthetic performance index; measurement of the energy absorbed by a single PSII reaction centre); ABS/RC (the light energy absorbed by the PSII antenna photon flux per active reaction centre), TR_0_/RC (total energy used to reduce QA by the unit reaction centre of PSII per energy captured by a single active RC), ET_0_/RC (rate of electron transport through a single RC), DI_0_/RC (nonphotochemical quenching per reaction centre of PSII; total dissipation of energy not captured by the RC in the form of heat, fluorescence and transfer to other systems), area – area over the curve between *F*_0_ and *F*_M_, corresponding to the size of the electron transport acceptor field of PSII, *Q*_p_ – photochemical extinction coefficient, and (1 − *V*_j_)/*V*_j_ – number of closed RCs in relation to their total quantity. Chlorophyll-induced fluorescence parameters were measured with a PAR-FluorPen FP 110D fluorimeter (Photon Systems Instruments, Drásov, Czech Republic).

#### Microbial toxicity

Each compound was dissolved in DMSO : H_2_O mixture (80% v/v) to achieve an initial solution of a concentration of 5000 mg dm^−3^. The initial mixtures were appropriately diluted in water to obtain the following concentrations: 4000, 3000, 2000, 1000, 500, 250, 50 mg dm^−3^. In addition, 80% (v/v) solution of DMSO in water was diluted similarly as samples, was tested as an abiotic control. All solutions were stored in the dark at 20–25 °C until use (for up to 7 days). Two bacterial strains, *Bacillus cereus* (Gram-positive bacteria) and *Pseudomonas putida* (Gram-negative bacteria) as well as *Candida albicans*, a member of yeast species, were chosen to evaluate the antimicrobial activity of analysed compounds. Each culture was transferred from agar plates into 50% TSB broth (Sigma Aldrich, Poland) with the optical density (OD_600_) of approx. 0.1. Minimum inhibitory concentration (MIC) as well as minimum bactericidal concentration (MBC) or minimum fungicidal concentration (MFC) values were determined in order to evaluate the antimicrobial activity of the analysed compounds. The procedure was performed in accordance with the European Committee on Antimicrobial Susceptibility Testing guidelines by means of micro-dilution method.^39^ Briefly, 50 mm^3^ of ILs solutions were transferred into the sterile 96-well plate in triplicates. Then, 200 mm^3^ of the bacterial suspension (approx. 2 × 10^4^ CFU cm^−3^) in 50% TSB medium with resazurin solution (40 μg cm^−3^) were added to the wells in order to obtain the final concentration of analysed compounds equal to 1000, 800, 600, 400, 200, 100, 50, 10 mg dm^−3^. The above-mentioned bacterial suspensions without the addition of analysed compounds was used as a biotic control, while ILs solution in 50% TBS medium with resazurin (40 μg cm^−3^) but without microorganisms was used as abiotic control. After 24 h of incubation at 30 °C, both MIC and MBC parameters were determined.

## Results and discussion

### Synthesis and identification

To obtain the designed ILs based on cinchona alkaloids, it was necessary to synthesize quaternary derivatives of both quinine and quinidine in the first step. 1-Alkylquininium bromides of high purity were obtained by the Menschutkin reaction using a homologous series of 1-bromoalkanes comprising 2 to 12 carbon atoms. DMSO was used as a solvent due to its ability to promote a reaction that follows the S_N_2 mechanism. Furthermore, due to the instability of quinine at elevated temperature, the process was carried out at 35 °C. The obtained bromides were subsequently purified by precipitation followed by double recrystallisation, according to the methodology described previously.^35^

**Fig. 1:**
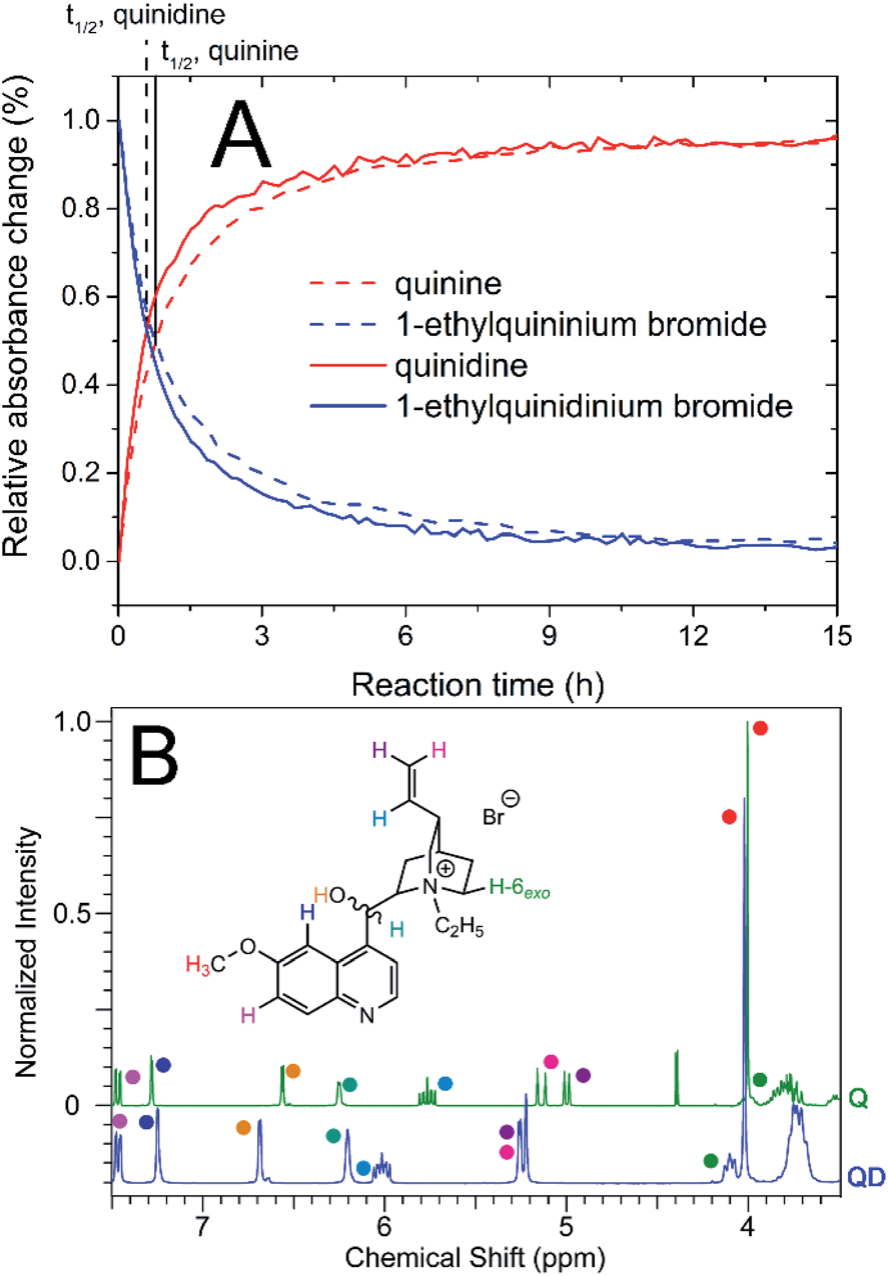
Kinetic curves for quinidine and quinine quaternizations with bromoethane (A) and ^1^H NMR spectra of both quaternary products in the chemical shift range 3.5 to 7.5 ppm (B); Q – product derived from quinine, QD – product derived from quinidine.

In addition, we developed an analogous method to obtain a source of cation derived from quinidine – 1-ethylquinidinium bromide. The compound was synthesized using identical reaction conditions (at 35 °C with DMSO as a solvent) and, as in the case of 1-ethylquininium bromide synthesis, we used 2-propanol as a recrystallisation solvent. Since the syntheses were performed using an FT-IR spectrometer *in situ* spectral acquisition, it was possible to determine that the conversion rates for both quinine and quinidine were sufficient (>95%) after 13 and 11 h, respectively. The comparison of kinetic curves is presented in Fig. 1A. In addition, on the basis of changes in absorbance at *ῡ* = 912 cm^−1^, which is a well-known signal originating from quinidine free base,^40^ we determined that the half-life of quinidine in the reaction system was approximately 20% shorter than that of quinine. Since the reaction conditions were the same for both alkaloids, it can be concluded that the chirality of the alkaloid affected the alkylation rate, albeit not substantially. Similar changes in the kinetics of various chemical processes due to the differences in chirality have been described previously.^41–43^

1-Ethylquinidinium bromide was obtained with a yield of 82%. On the basis of the nuclear magnetic resonance spectra obtained, it was possible to confirm that the chemical structure of the product was correct. The ^1^H and ^13^C NMR spectra of 1-ethylquinidinium bromide are disclosed in the ESI (ESI, Fig. S1 and S2†). We observed that in the ^1^H NMR spectra, several signals originating from hydrogen atoms in the 1-ethylquinidinium cation occurred with different chemical shifts compared to their counterparts in the 1-ethylquininium cation. As an example, the change in chirality caused significant deshielding of the protons in the vinyl group (H-10, H-11_*cis*_ and H-11_*trans*_, Δ*δ* ≤ 0.25 ppm) and the proton present in the hydroxymethylene group (OH, Δ*δ* ≤ 0.2). In contrast, the hydrogen atom H-2_*endo*_ in the 1-ethylquinidinium cation is shielded compared to its counterpart in the quinine-based cation: its signal occurred at *δ* = 0.96 ppm, while the respective signal from the 1-ethylquininium cation was observed at 1.36 ppm. In addition, hydrogen atoms in the quinoline group, which are not in the vicinity of chiral carbon atoms, were characterized by the same chemical shifts, independent of the chirality of the cation. The chemical shift changes described above were analogous to the well-known differences between signals originating from the free base forms of quinine and quinidine.^44^ However, it should be noted that the signals originating from the ethyl substituent also occurred at different chemical shifts depending on the chiral form of the cation.

**Fig. 2:**
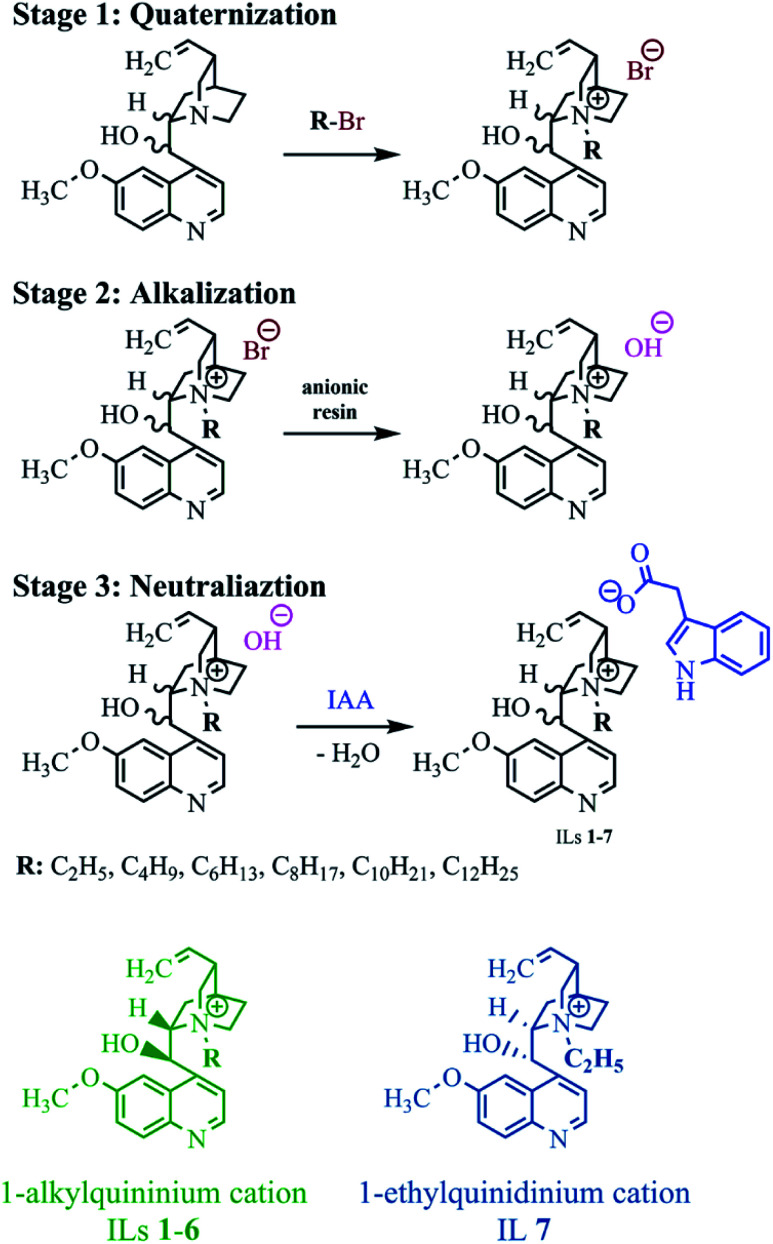
Synthesis of ILs with 1-alkylquininium (1–6) or 1-alkylquinidinium (7) cation and IAA anion.

As in the case of the previously described quinine-based aprotic ILs,^35^ the designed products with the indole-3-acetate anion were obtained in an two-step anion exchange reaction. The reactions are shown in Fig. 2. In the 2nd stage of the entire process, the obtained quaternary bromides were transformed into respective quaternary hydroxides *via* alkalization by contact with a bed of ion exchange resin. In the 3rd and final stage, the hydroxide obtained was immediately neutralized with indole-3-acetic acid, yielding the designed IL and water. All syntheses were performed using naturally derived ethanol, which could be reused after the process.

The results of the syntheses are presented in Table 1. As with previous syntheses using an alkaline ion exchange resin,^32,35,45^ the products were obtained with very high yields (99–100%) due to the very high efficiency of the adopted method. Neither the length of the alkyl substituent in the 1-alkylquininium cation nor the absolute configuration of the cation affected the efficiency of the ion exchange reaction.

**Table 1:** Synthesized ILs with a quinine (1–6) or quinidine-based (7) cation and IAA anion

IL	Cation	R	Yield [%]	*T*_g_ a [°C]	*T*_5%_ b [°C]	*T*_50%_ c [°C]
1	Quininium	C_2_H_5_	99	—	218	272
2	Quininium	C_4_H_9_	99	47	205	272
3	Quininium	C_6_H_13_	100	60	215	293
4	Quininium	C_8_H_17_	99	—	217	304
5	Quininium	C_10_H_21_	99	40	220	321
6	Quininium	C_12_H_25_	100	49	220	329
7	Quinidinium	C_2_H_5_	99	—	218	271

a Glass transition temperature.

b Temperature of the decomposition of 5% of the sample.

c Temperature of the decomposition of 50% of the sample.

All products obtained were amorphous, glassy solids at 25 °C. A similar state was observed for the majority of other quinine-based ILs.^33,35^ This means that the cation based on cinchona alkaloid influenced the physical state and the viscosity of the obtained products. These phenomena may be partly attributed to the presence of the hydroxyl moiety in the alkaloid-based cation, which is a donor of particularly strong hydrogen bonds^46^ that are only partially disrupted by the introduction of an organic anion.^33^ The analysis of ^1^H NMR, ^13^C NMR and FT-IR spectra confirmed that the chemical structures of the obtained ILs were correct. The spectra are provided in the ESI (Fig. S3–S5, S7–S9, S11–S13, S15–S17, S19–S21, S23–S25 and S27–S29†). In the ^13^C NMR spectra, characteristic signals from atoms present in the indole-3-acetate anion were identified, including signals from the methylene group (*δ* = 35 ppm), indole aromatic ring (*δ* = 110–140 ppm) and carboxylate group (*δ* = 175 ppm). The corresponding signals were also found in the ^1^H NMR spectra, *e.g.*, the signals for the methylene moiety (*δ* = 3.4 ppm), for hydrogen atoms present in the indole group, and the characteristic signal at approximately 11.0 ppm from the NH group. The presence of these signals confirmed the full, successful exchange of the bromide anion for IAA-derived ions. It should be emphasized that the length of the alkyl substituent did not have an obvious impact on the chemical shifts or wavenumbers of signals derived from anions.

In addition, we attempted to synthesise a protic IL by combining quinine with IAA in the equimolar ratio. However, further spectral analysis of the obtained product confirmed that the difference in acidity between both reagents is too low, and quinine cannot be protonated by IAA to any significant degree. Based on the NMR spectra (Fig. S31–S33, ESI†), we confirmed the lack of ionic bond between unmodified quinine and IAA following their homogenization. The signals observed corresponded to quinine in the free base form; moreover, all signals from the IAA structure in the NMR spectra were deshielded compared to their counterparts in the spectra of ILs 1–7. This indicates that the quinine–IAA system is a binary mixture and not a protic IL. We have nevertheless applied the resulting system as a reference in further studies.

### Thermal analysis

The results of differential scanning calorimetry and thermogravimetric analysis of the obtained ILs are summarized in Table 1. In addition, thermogravimetric analysis plots for all ILs are presented in ESI (Fig. S6, S10, S14, S18, S22, S26 and S30†). Unlike the majority of products with quinine-based cation and bis(trifluoromethylsulfonyl)imide anions,^33^ none of the compounds exhibited melting points or crystallisation temperatures in the temperature range 100 to 120 °C. However, glass transition temperatures were observed for 4 of the obtained ILs: 2, 3, 5 and 6. These temperatures ranged from 40 °C (5) to 60 °C (3) and were poorly correlated with the lengths of the alkyl substituents present in the 1-alkylquininium cations. The results of the analyses conducted also indicated that the indole-3-acetates with 1-butylquininium (2) and 1-dodecylquininium (6) were characterized by glass transition temperatures that were 11 and 10 °C lower, respectively, compared to those of indole-3-butyrates containing the same cations.^35^ Moreover, glass transition temperature similar to that recorded for 3 (56 °C) was also observed for the IAA-quinine binary mixture.

It should also be emphasized that, among the tested compounds, products containing a cation with an ethyl (1, 7) or octyl (4) substituent did not undergo any phase transition in the temperature range analysed. This indicates that cations derived from cinchona alkaloids possessing the substituents mentioned above affected the structures of the ILs to such a degree that the transition from the glassy state to the liquid state was hindered and resided outside of the studied temperature range.

Based on the results of thermogravimetric analysis, decomposition temperatures for 5% of the sample mass (*T*_5%_) and half of the sample mass (*T*_50%_) were determined. Despite significant changes in the chemical structures of the cations, the *T*_5%_ values ranged from 205 °C (2) to 220 °C (5 and 6). Similar levels of thermal stability were also registered for other quinine-derived ILs.^33,35^ The relatively small distribution of *T*_5%_ values indicates that in the initial stages of degradation of quinine and quinidine-derived indole-3-acetates, the groups present in the anion primarily undergo degradation. However, the range of *T*_50%_ values was significantly broader and extended from 271 (8) to 329 °C (6). Therefore, it can be concluded that the IL 6 with the longest alkyl substituent was the most thermally stable of the compounds analysed and that the stability was correlated with the length of the alkyl chain present in the cation of the ionic compound. It should be emphasized that the difference between the *T*_50%_ values for the last and first elements of the homologous series (57 °C) was much larger than that for the homologous series comprising 1-alkyl-1-methylpiperidinium,^25^ alkyl(2-hydroxyethyl)dimethylammonium^32^ or alkyl[2-(2-hydroxyethoxy)ethyl]dimethylammonium^47,48^ cations. The obtained values confirmed that ILs with cations derived from cinchona alkaloids have higher thermal stability than compounds containing a synthetic cation, and this indicates a different route for thermal degradation of cinchona alkaloids.

### Solubility

The results of the solubility tests are summarized in Table 2. As with the previously described ILs with a 1-alkylquininium cation and an organic anion, all obtained indole-3-acetates 1–7 were characterized by ready solubility in most of the analysed organic solvents exhibiting high (methanol, DMSO, acetone) or medium (2-propanol, chloroform) polarity. It should be noted that only IL 7 with a cation derived from quinidine exhibited low solubility in chloroform. Moreover, only in acetonitrile did the solubility depend on the length of the alkyl substituent in the 1-alkylquininium cation. Both compounds containing the ethyl substituent (1 and 7) exhibited weak affinities for this solvent and the IL with the butyl substituent (2) exhibited medium solubility, while the compounds with a hexyl or longer substituent in the cation (3–6) were readily soluble in acetonitrile. In contrast to the previously analysed salts with the 1-alkylquininium cation, the length of the alkyl substituent did not affect the solubility of the tested compounds in ethyl acetate, in which none of the tested indole-3-acetates exhibited noticeable solubility. As with the previously analysed ILs with 1-alkylquininium cations and indole-3-butyrate, theophyllinate and (*S*)-mandelate anions,^35^ the quinine and quinidine derivatives containing indole-3-acetate anions did not show solubility in the least polar solvents toluene and hexane.

**Table 2:** Solubility of synthesized ILs

IL	A^a^	B	C	D	E	F	G	H	I	J
	9.0^b^	6.6	6.5	6.2	5.1	4.3	4.3	4.1	2.3	0.0
1	−	+	+	−	+	+	−	+	−	−
2	−	+	+	±	+	+	−	+	−	−
3	−	+	+	+	+	+	−	+	−	−
4	−	+	+	+	+	+	−	+	−	−
5	−	+	+	+	+	+	−	+	−	−
6	−	+	+	+	+	+	−	+	−	−
7	−	+	+	−	+	+	−	−	−	−

a A – water, B – methanol, C – DMSO, D – acetonitrile, E − acetone, F – 2-propanol, G – ethyl acetate, H – chloroform, I – toluene, J – hexane.

b Snyder polarity index; “+”, ready solubility; “±”, limited solubility; “−”, low solubility.

The results of the solubility analysis indicated that the tested ILs with 1-alkylquininium and 1-ethylquinidinium cations were characterized by low solubility in the most polar of the tested solvents – water. This results from a combination of two highly hydrophobic ions in the structure of one chemical compound. It should be noted that not all quinine-based ILs are characterized by low water solubility; in a previously reported study, 1-butylquininium salts with theophyllinate and (*S*)-mandelate anions were characterized by water solubility exceeding 100 g per 1 dm^3^ of solvent. However, low affinity for water does not prevent the use of compounds 1–7 as plant growth hormones in the form of aqueous solutions, since natural auxins (including indole-3-acetic acid) exhibit satisfactory biological activity at very low concentrations (0.01–1 mg dm^−3^).^14,18^

### Influence on plant cultivation

**Fig. 3:**
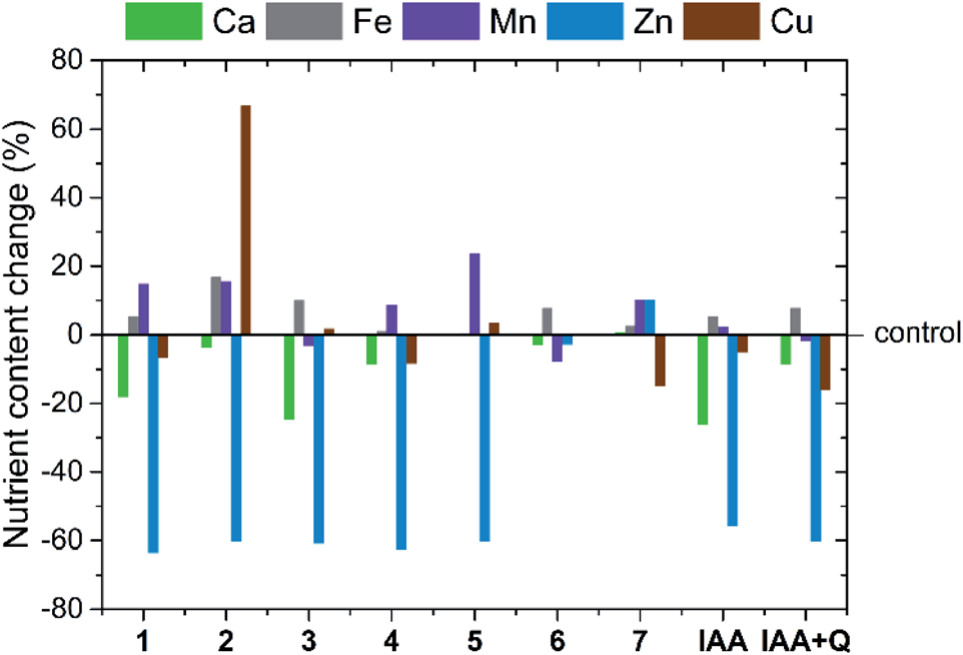
Change of the average content of selected micronutrients and macronutrients in lettuce plants grown with nutrient solution containing ILs 1–7, IAA or IAA-quinine binary mixture (IAA + Q) in comparison to control plants.

The application of natural auxins to numerous species of crop plants results not only in a change in the growth and development of shoots and roots but may also be beneficial in improving stress tolerance towards unfavourable factors^49–51^ and affecting the uptake and transport of nutrients within the plant.^18,52^ This last aspect is particularly important, since the uptake of nutrients by plants is not only related to their proper development but, in the case of edible crops, it also affects their nutritional value. Therefore, we thoroughly analysed the effect of auxin-based ILs on lettuce plants, with particular emphasis on changes in the chemical composition of the plant tissues. The chosen concentration of the tested compounds, 0.5 mg dm^−3^ in the nutrient solution, made it possible to observe significant changes in the content of particular nutrients while imparting no toxic effects on the plants. Moreover, IAA in the acid form, as well as the obtained binary mixture of IAA and quinine were tested as reference systems at the same concentration. The obtained results for selected macro- and micronutrients, expressed as percentage change with respect to the results recorded for the control plants, are summarized in Fig. 3. The summary for all analysed nutrients as well as numerical data are provided in ESI, Fig. S34 and Tables S1, S2,† respectively.

Based on the data presented in Fig. 3, it can be concluded that the very minor addition of an auxin-based IL or a reference substance caused a statistically significant difference in the uptake of at least one of the following nutrients: calcium, iron, manganese, zinc or copper. In contrast, no significant differences were generally observed in the uptake of nitrogen, phosphorus, potassium, magnesium and sodium in relation to the respective controls.

Despite the fact that the application of IAA at similar concentrations to other species of plants (*i.e.*, *Medicago sativa* L.^53^ or *Capsicum annuum* L.^18^) usually caused an increase in calcium uptake, we observed a clear negative outcome for the uptake of this nutrient. In fact, the calcium content was 26% lower than that in the control plants. Calcium deficiency is a known cause of physiological disorders in lettuce and other crops (known as a tip burn);^54^ therefore, factors limiting the content of this nutrient may be a direct cause of adverse effects. In addition, the application of the reference substance also caused more than a twofold reduction in zinc content (56%) in the aboveground parts of lettuce plants. Such a significant decrease in zinc uptake reduces the dietary value of the plants.^55^

It should be stressed, however, that in the majority of cases the replacement of IAA by ILs with cations derived from cinchona alkaloids mitigated the abovementioned negative effects on calcium uptake: apart from 1 and 3, which caused decreases of 18 and 25%, respectively, the other compounds with a cation derived from quinine resulted in values that did not show significant differences compared to the control plants. Moreover, it is worth mentioning that for 4 of the obtained growth regulators (2, 5, 6 and 7), the change in calcium content in comparison to the control did not exceed ±5%. We can thus conclude that despite the presence of an IAA-derived anion in the medium at an equivalent concentration, the unique effect of the alkaloid cations counteracted the negative influence of auxin on calcium uptake. It is also worth noting that the addition of quinine free base in the IAA + Q reference system resulted in a similar change. However, the majority of the tested compounds (ILs 1–5) exhibited a negative effect on zinc content in plants as compared with the reference substances (from −63 to −60%). We discovered that this unfavourable phenomenon could be successfully addressed by the introduction of a suitably long alkyl substituent (IL 6, change −3%) or by alteration of the optical activity of the cation (IL 7, change +10%). In addition, it is noteworthy that the introduction of the cation 1-ethylquinidinium (7) instead of 1-ethylquininium (1) caused a radical change in zinc content in plant dry matter, by as much as 73.6% of the content of this micronutrient in control plants.

The action of IAA had little effect on the contents of manganese, iron and copper in lettuce plants. However, a significant increase in the content of the above elements was observed with the application of some ILs. For example, the addition of IL 5 to the nutrient solution resulted in a 24% increase in plant manganese content relative to control plants. Similarly, two ILs with 1-alkylquininium cations containing the shortest alkyl substituents (1 and 2) caused an approximately 15% increase in manganese content. The above differences were statistically significant. We also observed that the addition of 1-butylquininium indole-3-acetate (2) resulted in a considerable increase in the iron content (17%) and copper content (up to 67%) of treated plants. Such a major change in the content of one of these micronutrients indicates that the efficiency of uptake of specific mineral substances is influenced by unique biochemical interactions with the cation of an IL. This effect does not occur for other known auxin-based ILs,^32^ although similar changes in copper uptake were observed in annual pepper plants treated with IAA at a concentration of 1 mg dm^−3^.^18^ However, in other cases, auxin addition resulted in a decrease in copper content in plant leaves and stems.^56,57^ It should be stressed that 2 was the only IL exhibiting such an effect among the products tested; in the case of the other compounds, generally no statistically significant differences were observed in the contents of iron or copper compared to the control.

The appearance of the abovementioned changes in the chemical compositions of plants proves that the introduction of ILs containing a cation derived from a cinchona alkaloid eliminated the negative effect of IAA on the uptake of calcium and zinc and, in individual cases, stimulated the uptake of other micronutrients by the plant. Furthermore, during the analysis of the biological activity of the compounds from the homologous series with the 1-alkylquininium cation (1–6), we determined that the changes in nutrient content exhibited no correlation with the lengths of the alkyl substituents. Therefore, it can be concluded that stimulation or inhibition of the uptake of specific nutrients by lettuce is influenced by the unique interactions with individual ionic liquid cations.

Basic biometric measurements, including yield, dry matter content (%DM) and relative water content (RWC), as well as detailed evaluation of physiological parameters, were performed for all test objects. The results of the analyses are provided in Tables S3–S5 in the ESI.† The yield of plants treated with ILs 1–7 and the reference substance ranged from 136.1 (6) to 153.3 g per plant (1); the recorded values showed no significant differences with respect to the control objects (149.3 g per plant). Moreover, the values of the %DM and RWC parameters ranged from 4.6 (5) to 4.9% (2) and from 72.7 (3) to 81.4% (1), respectively. The studied parameters did not show differences in comparison to those of the control objects and were within the standard range for healthy lettuce plants.^58,59^ Thus, it can be concluded that the addition of ILs with cations based on alkaloids did not cause any noticeable phytotoxic effects for the experimental objects.

This conclusion is also supported by the fact that no significant changes in physiological parameters indicative of stress conditions were observed in the plants studied (Tables S4 and S5, ESI†). Since the magnitude of chlorophyll fluorescence is inversely proportional to the intensity of photosynthesis, such measurements can be a valuable tool for assessing the functioning of the photosynthetic apparatus as well as the health and vitality of the plant.^60^ For example, the highest values of fluorescence parameters, *F*_0_, *F*_M_ and *F*_V_, were observed for control plants, while in the case of objects treated with the majority of ILs and IAA, these parameters exhibited significantly lower values. Moreover, the calculated maximum quantum yield of photosystem II (PSII) in the dark (*F*_V_/*F*_M_),^61^ potential activity of PS II (*F*_V_/*F*_0_)^62^ and photochemical quenching (qP) did not differ significantly between the control and experimental objects. This confirmed that the addition of ILs did not cause adverse physiological changes in plants. Only in the case of compound 3 were unfavourable changes in physiological parameters related to the efficiency of PSII reaction centres: different values of ABS/RC, TR_O_/RC and ET_O_/RC were observed in relation to the control. However, these differences did not translate directly into a decrease in yield or a change in the chemical composition of the aboveground parts.

### Microbial toxicity

High microbial toxicity, especially towards Gram-positive bacteria, is a characteristic and well-known property of quaternary ammonium salts with amphiphilic structures, including ILs with alkyl-substituted cations and organic anions.^63–66^ This phenomenon is associated with an increased tendency to incorporate into and disrupt the structure of the cell membrane of the microorganism, as well as to induce cell autolysis resulting from the degradation of intracellular proteins and nucleic acids.^66^ Therefore, we performed a toxicity analysis of the obtained ILs with quaternary cations derived from quinine or quinidine towards 3 model microorganisms. The results are presented in Table 3. The tested ILs exhibited typical properties for a homologous series of quaternary ammonium compounds, for which an increase in the number of carbon atoms in the substituent above 8 resulted in an increase in toxicity. Detailed analysis of the obtained data revealed that the highest antimicrobial activity was determined for ILs 4–6 containing the longest alkyl substituents (from octyl to dodecyl), and MIC values were below 50 ppm for all species (except 4 for *P. putida*, which was the least susceptible of the tested organisms). In contrast, negligible or low toxicity was recorded for compounds involving an alkyl substituent shorter than octyl (1–3 and 7). Due to their low amphiphilicity, these products were characterised by MIC and MBC values towards Gram-negative *P. putida* and *C. albicans* (yeast) that were similar to those observed for the respective DMSO controls. However, the same ILs (1–3, 7) exhibited marginally higher toxicity towards Gram-positive *B. cereus* – the most susceptible of the tested organisms – compared to the DMSO control.

**Table 3:** Microbial toxicity of the obtained ILs 1–8 and reference substances: quinine (Q), quinine–IAA binary mixture (IAA + Q), 1-octylquininium bromide ([Q-C_8_][Br]), potassium indole-3-acetate ([K][IAA])

Compound	Microbial toxicity [μg g^−1^]								
	*Bacillus cereus*			*Pseudomonas putida*			*Candida albicans*		
	MIC	MBC	Toxicity classification^a^	MIC	MBC	Toxicity classification	MIC	MFC	Toxicity classification
1	100	400	PH	400	600	PH	100	400	PH
2	200	600	PH	600	600	PH	100	400	PH
3	100	400	PH	400	600	PH	100	400	PH
4	<10	50	ST	400	600	PH	10	100	ST
5	<10	<10	MT	50	100	ST	<10	50	ST
6	<10	<10	MT	50	100	ST	<10	<10	MT
7	400	600	PH	400	600	PH	100	400	PH
Q	200	400	PH	400	600	PH	100	400	PH
IAA + Q	100	400	PH	600	600	PH	100	400	PH
[Q-C_8_][Br]	<10	50	ST	400	600	PH	50	100	ST
[K][IAA]	400	800	PH	400	600	PH	200	400	PH
DMSO (control)	400	800	PH	400	600	PH	200	400	PH

a Toxicity scale: <0.01 mg L^−1^ supertoxic; 0.01–0.1 mg L^−1^ extremely toxic; 0.1–1.0 mg L^−1^ highly toxic; 1–10 mg L^−1^ moderately toxic; 10–100 mg L^−1^ slightly toxic; 100–1000 mg L^−1^ practically harmless; >1000 mg L^−1^ relatively harmless.

According to the classification proposed previously by Passino and Smith,^67^ most of the synthesized cinchona-based ILs may be described as practically harmless towards all tested microorganisms since they exhibited biological activity at concentrations ranging from 100 to 1000 mg dm^−3^. It should be noted that the obtained results are in line with the toxicity data provided for other quinine-based quaternary salts that did not contain long alkyl substituents.^34^ However, due to the enhanced amphiphilicity, compound 4 was assessed as slightly toxic (10–100 mg dm^−3^) towards *B. cereus* and *C. albicans*, and compounds 5 and 6 expressed slight toxicity towards *P. putida* and moderate toxicity (1–10 mg dm^−3^) towards *B. cereus*. IL 6 was also moderately toxic towards *C*. *albicans*. In general, the tested derivatives exhibited low environmental risk on the basis of simple tests with model microorganisms, and since they are designed to be applied at a concentration of 0.5 mg dm^−3^, which is significantly below the threshold of their microbial toxicity, we believe that the likelihood of the occurrence of toxic effects on microorganisms present in the environment is relatively low. However, more adequate environmental studies are needed before their introduction to commercial markets.

## Conclusions

In the present study, new ILs derived from IAA with quinine and quinidine-based cations were successfully synthesized; the majority of the synthesized products constituted a homologous series comprising 1-alkylquininium cations with substituents of varying length (from 2 to 12 carbon atoms). The ion exchange products were obtained in the theoretical yields without the use of hazardous substrates or media and without the need for additional purification of the obtained ILs. These features indicate the high potential for application of our proposed method. The chemical structures of ILs were fully confirmed by NMR analyses. Considering that auxins are plant hormones showing activity even at doses below 1 mg dm^−3^, the low solubility of the obtained ILs in water is not an obstacle for the preparation of functional formulations. We also discovered that only ILs containing a substituent longer than hexyl in the cation showed any noticeable toxicity towards microorganisms. Nevertheless, the application concentration of the compounds studied is sufficiently low as to mitigate the observed toxic effect. It should be mentioned that at a concentration of 0.5 mg dm^−3^, the obtained IAA derivatives did not show toxic effects against lettuce plants, which was confirmed by biometric measurements and chlorophyll fluorescence analyses. Moreover, ILs 6 and 7 were able to positively neutralize the negative effects of IAA in the acid form: the reduction of zinc and calcium content in plant dry matter. It should also be noted that the use of other compounds (in particular IL 2) had a positive effect on the contents of micronutrients that are important aspects of the human diet: manganese, iron and copper. This means that the strategy of converting IAA into ILs may be crucial in the cultivation of plants with specific nutrient contents.

## Author contributions

Tomasz Rzemieniecki: conceptualization, methodology, investigation, data curation, visualization, validation, writing – original draft, writing – review & editing. Tomasz Kleiber: methodology, investigation, data curation, formal analysis, resources. Juliusz Pernak: conceptualization, funding acquisition, resources, supervision.

## Conflicts of interest

There are no conflicts to declare.

## Supplementary Material

RA-011-D1RA04805H-s001
