# Supplementary material for: Naturally based ionic liquids with indole-3-acetate anions and cations derived from cinchona alkaloids
Source: RSC Adv. 2021 Aug 13;11(44):27530–40. doi: 10.1039/d1ra04805h (PMC9037897; doi:10.1039/d1ra04805h)
Supplement: RA-011-D1RA04805H-s001 [file RA-011-D1RA04805H-s001.pdf]

Electronic Supplementary Material (ESI) for RSC Advances.
This journal is © The Royal Society of Chemistry 2021

ELECTRONIC SUPPORTING INFORMATION

Naturally based ionic liquids with indole-3-acetate anions and cations derived from cinchona alkaloids

Tomasz Rzemieniecki,[†] Tomasz Kleiber [‡] and Juliusz Pernak[†]*

[†] Department of Chemical Technology, Poznan University of Technology, Berdychowo 4, Poznan 60-965, Poland

[‡] Department of Agronomy, Horticulture and Bioengineering, Poznan University of Life Sciences, Zgorzelecka 4, Poznan 60-198, Poland

* Corresponding author at: Poznan University of Technology, Berdychowo 4, Poznan 60-965, Poland; Tel.: +48 616653581. E-mail: juliusz.pernak@put.poznan.pl

Number of pages: 37

Number of tables: 5

Number of figures: 34

1. IDENTIFICATION OF COMPOUNDS

^{1}H NMR (400 MHz, DMSO-*d*$_6$) δ [ppm] = 0.90–1.02 (m, **H-7$_{endo}$**) 1.52 (t, *J* = 6.7 Hz, 3H, **N$^+$–CH$_2$–CH$_3$**), 1.71–1.88 (m, 2H, **H-5$_{endo}$**, **H-5$_{exo}$**), 1.89–1.94 (m, 1H, **H-4**), 2.26 (t, *J* = 11,3 Hz, 1H, **H-7$_{exo}$**), 2.73–2.82 (m, 1H, **H-3**), 3.40–3.50 (m, 1H, **H-6$_{endo}$**), 3.62–3.83 (m, 5H, **H-2$_{endo}$**, **H-2$_{exo}$**, **N$^+$–CH$_2$–CH$_3$**, **H-8**) 4.02 (s, 3H, **H-12**), 4.10 (t, *J* = 10.2 Hz, 1H, **H-6$_{exo}$**), 5.21–5.28 (m, 2H, **H-11-*trans***, **H-11-*cis***), 5.95–6.08 (m, 1H, **H-10**), 6.20 (s, 1H, **H-9**), 6.69 (d, *J* = 3.0 Hz, 1H, **OH**) 7.22–7.27 (d, *J* = 1.3 Hz, 1H, **H-5'**), 7.47 (dd, $J_{1,2}$ = 1.9 Hz, $J_{1,3}$ = 9.1 Hz, 1H, **H-7'**), 7.72 (d, *J* = 4.5 Hz, 1H, **H-3'**), 7.99 (d, *J* = 9.2 Hz, 1H, **H-8'**), 8.79 (d, *J* = 4.3 Hz, 1H, **H-2'**).

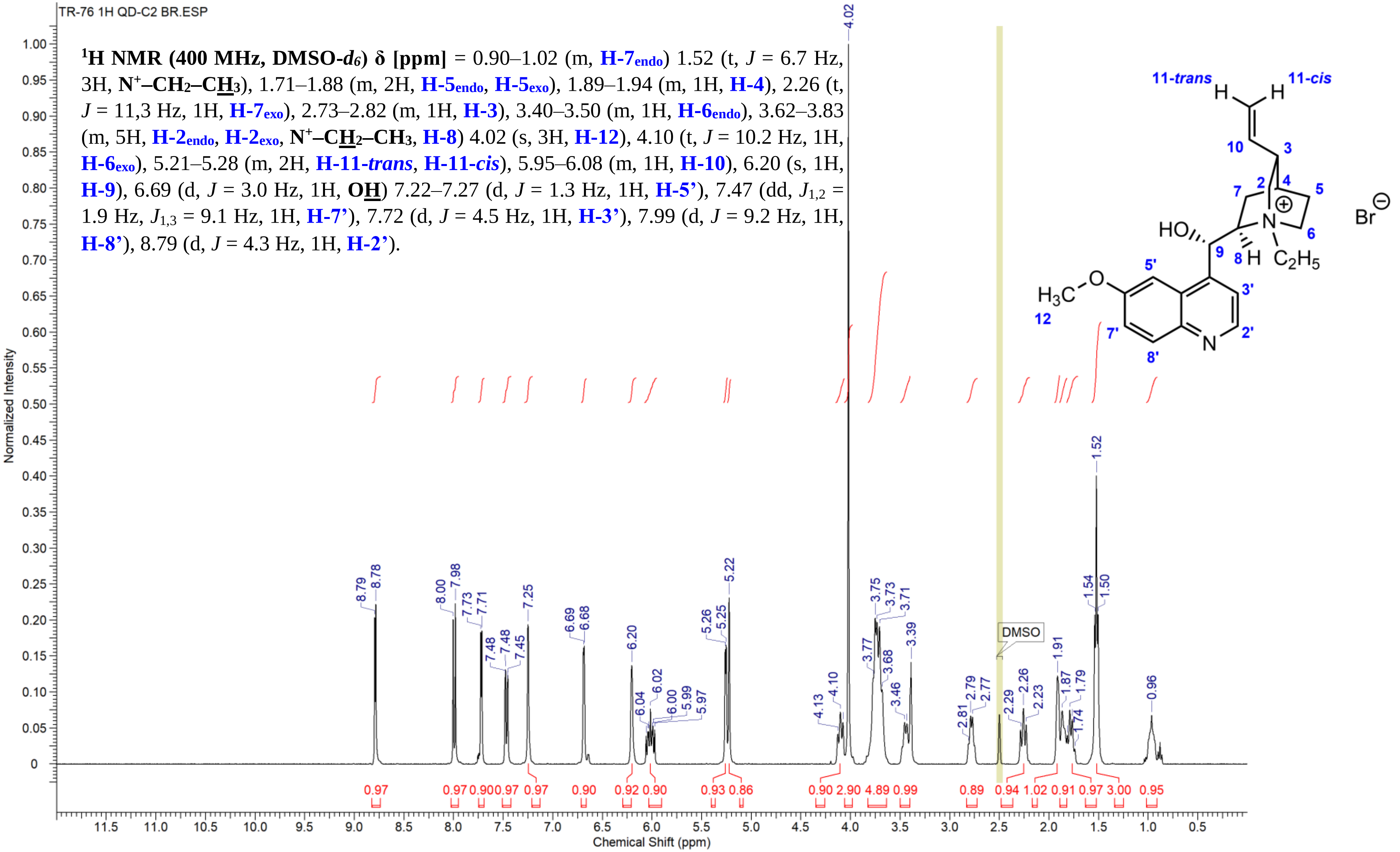

Figure S1. ^{1}H NMR spectrum of 1-ethylquinidinium bromide

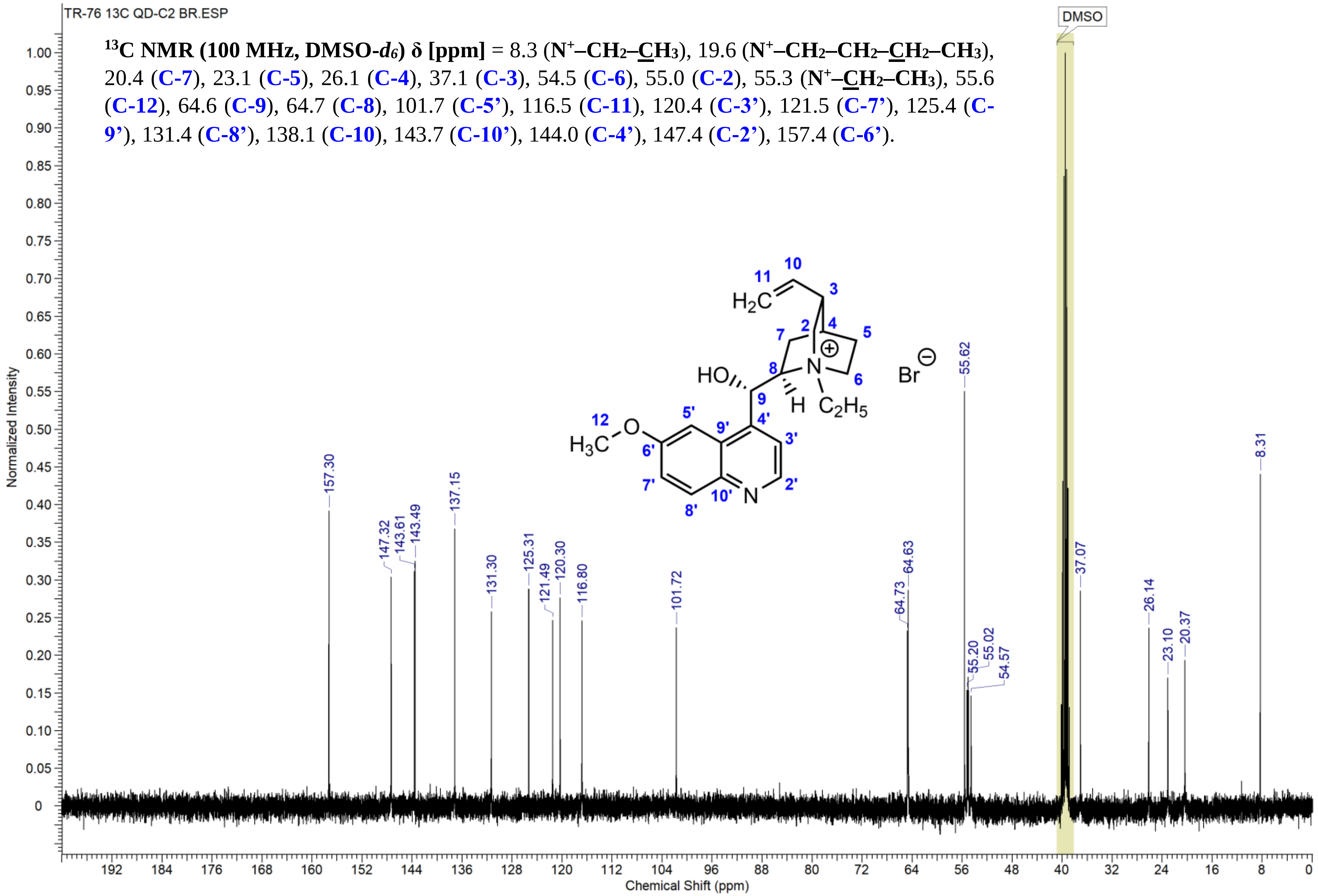

Figure S2. ^{13}C NMR spectrum of 1-ethylquinidinium bromide

^{1}H NMR (400 MHz, DMSO-d_6) δ [ppm] = 1.25 (t, J = 7.1 Hz, 3H, **N$^+$–CH$_2$–C<u>H</u>$_3$**), 1.41 (t, J = 11.7 Hz, 1H, **H-7$_{endo}$**), 1.72–1.82 (m, 1H, **H-5$_{endo}$**), 1.90–1.96 (m, 1H, **H-4**), 2.07–2.20 (m, 2H, **H-5$_{exo}$**, **H-7$_{exo}$**), 2.62–2.72 (m, 1H, **H-3**), 3.15–3.24 (m, 1H, **H-2$_{endo}$**), 3.25–3.33 (m, 1H, **H-6$_{endo}$**), 3.41 (s, 2H, **H-A**), 3.50 (t, J = 11.5 Hz, 1H, **H-2$_{exo}$**), 3.68–3.89 (m, 3H, **N$^+$–C<u>H</u>$_2$–(CH$_2$)$_2$–CH$_3$**, **H-8**), 3.92 (s, 3H, **H-12**), 4.14 (t, J = 11.1 Hz, 1H, **H-6$_{exo}$**), 5.00 (d, J = 10.5 Hz, 1H, **H-11-*trans***), 5.11 (d, J = 17.4 Hz, 1H, **H-11-*cis***), 5.69–5.81 (m, 1H, **H-10**), 6.51 (s, 1H, **H-9**), 6.88 (ddd, $J_{1,2}$ = 1.0 Hz, $J_{1,3}$ = 6.8 Hz, $J_{1,5}$ = 7.8 Hz, 1H, **H-E**), 7.01 (ddd, $J_{1,2}$ = 1.2 Hz, $J_{1,3}$ = 7.1 Hz, $J_{1,5}$ = 8.0 Hz, 1H, **H-F**), 7.11 (d, J = 2.1 Hz, 1H, **H-G**), 7.32 (d, J = 8.0 Hz, 1H, **H-C**) 7.33 (d, J = 2.7 Hz, 1H, **H-5'**), 7.46 (dd, $J_{1,2}$ = 2.6 Hz, $J_{1,3}$ = 9.3 Hz, 1H, **H-7'**), 7.52 (d, J = 7.8 Hz, 1H, **H-D**), 7.69 (d, J = 4.6 Hz, 1H, **H-3'**), 8.00 (d, J = 9.2 Hz, 1H, **H-8'**), 8.76 (d, J = 4.4 Hz, 1H, **H-2'**), 11.02 (s, 1H, **H-K**).

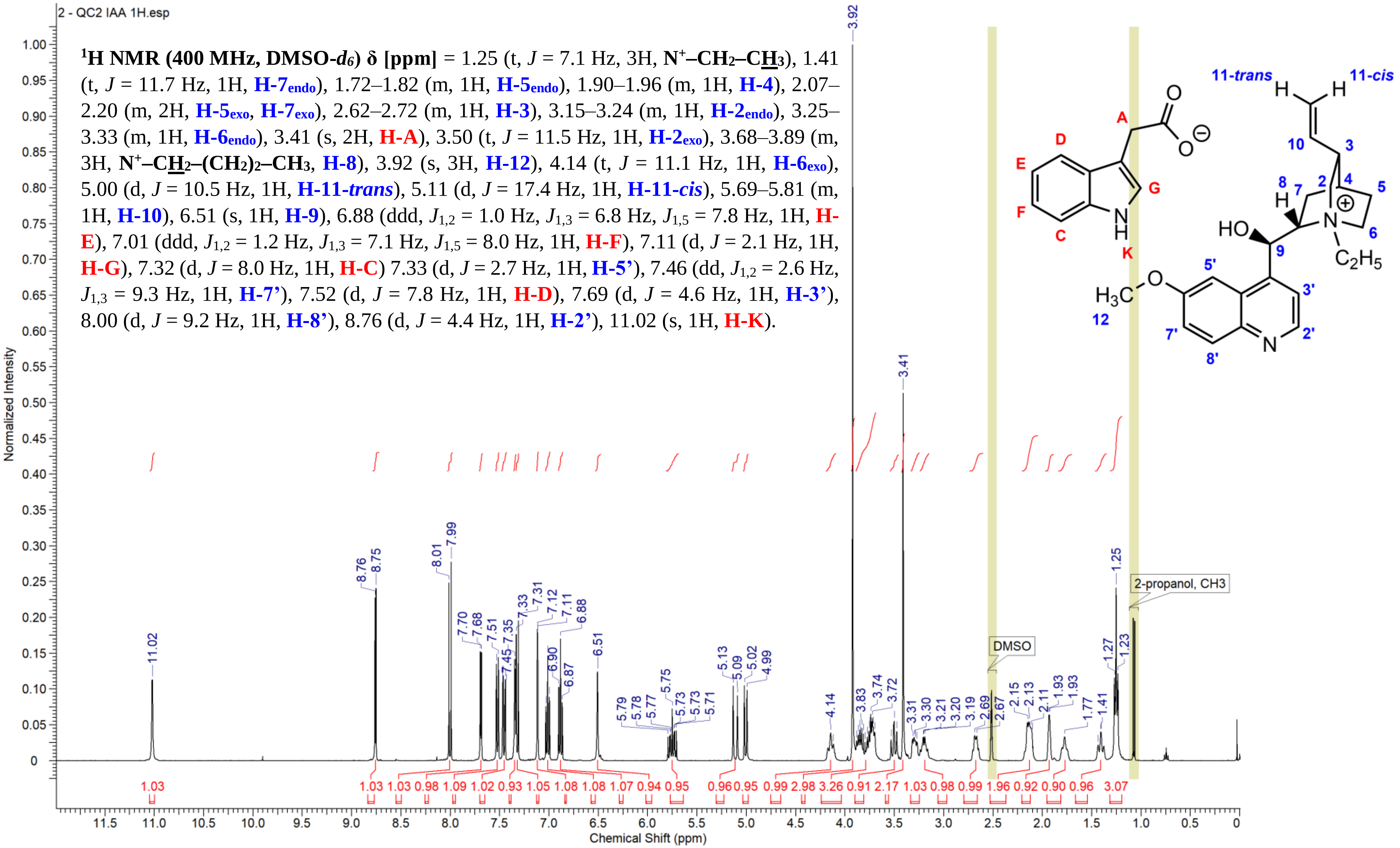

Figure S3. ^{1}H NMR spectrum of 1-ethylquininium indole-3-acetate (**1**)

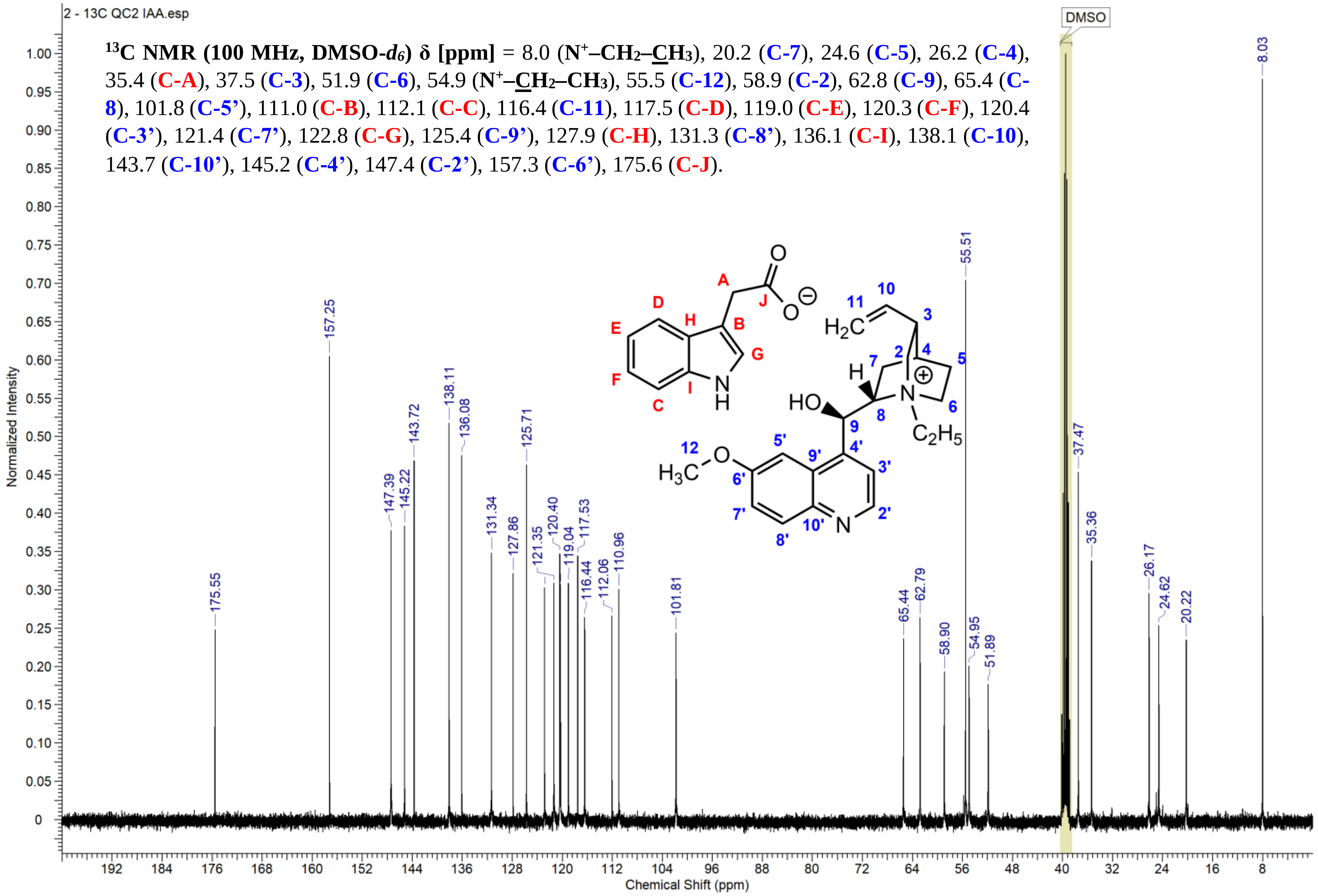

Figure S4. ^{13}C NMR spectrum of 1-ethylquininium indole-3-acetate (**1**)

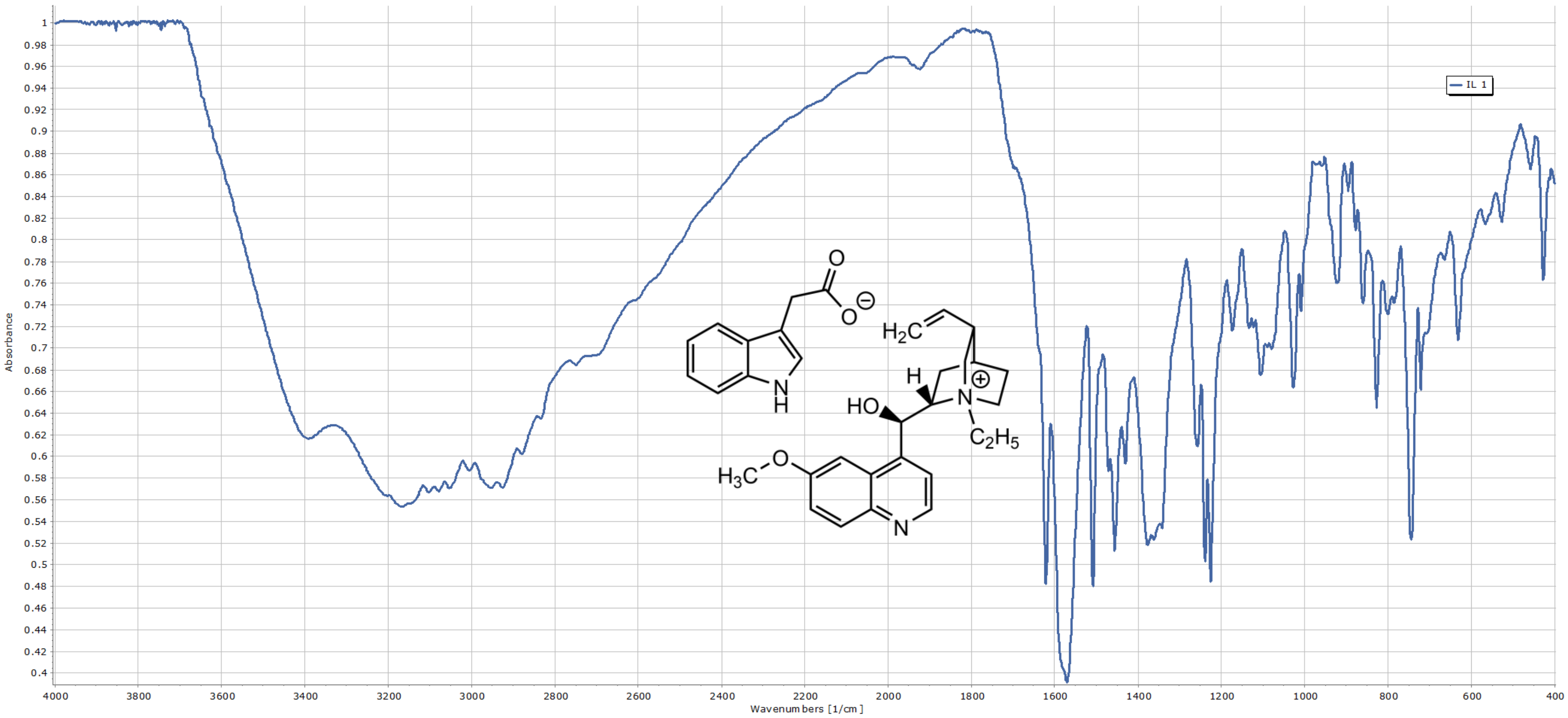

IR (KBr disc) ν [cm^{-1}] = 3392, 3169, 3102, 3078, 3053, 3005, 2952, 2925, 2879, 2834, 2749, 1621, 1570, 1508, 1456, 1430, 1377, 1362, 1342, 1258, 1239, 1226, 1174, 1133, 1105, 1079, 1027, 1008, 959, 924, 895, 860, 827, 800, 744, 721, 664, 633, 566, 527, 457, 427.

Figure S5. FT-IR spectrum of 1-ethylquininium indole-3-acetate (**1**)

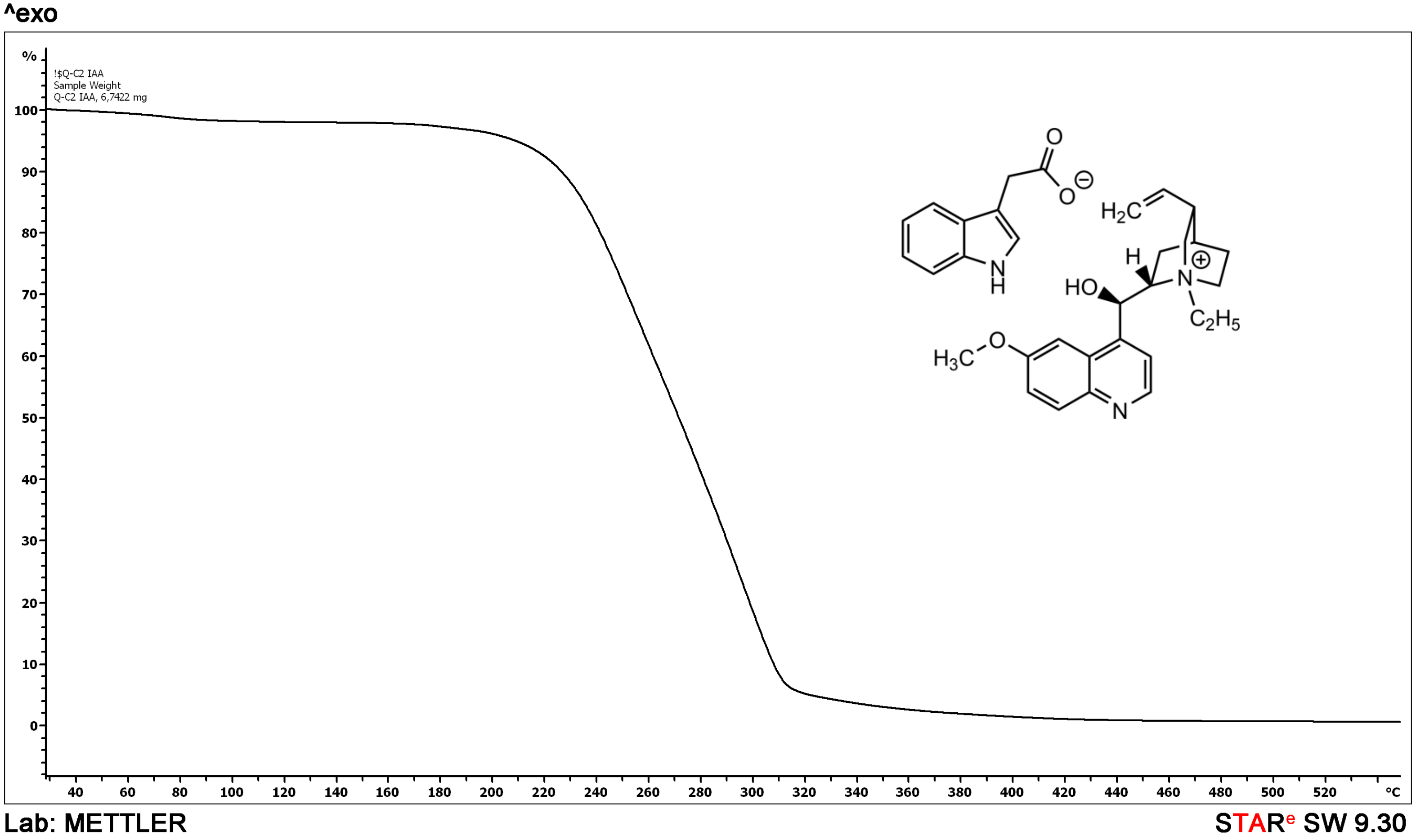

Figure S6. TGA plot of 1-ethylquininium indole-3-acetate (**1**)

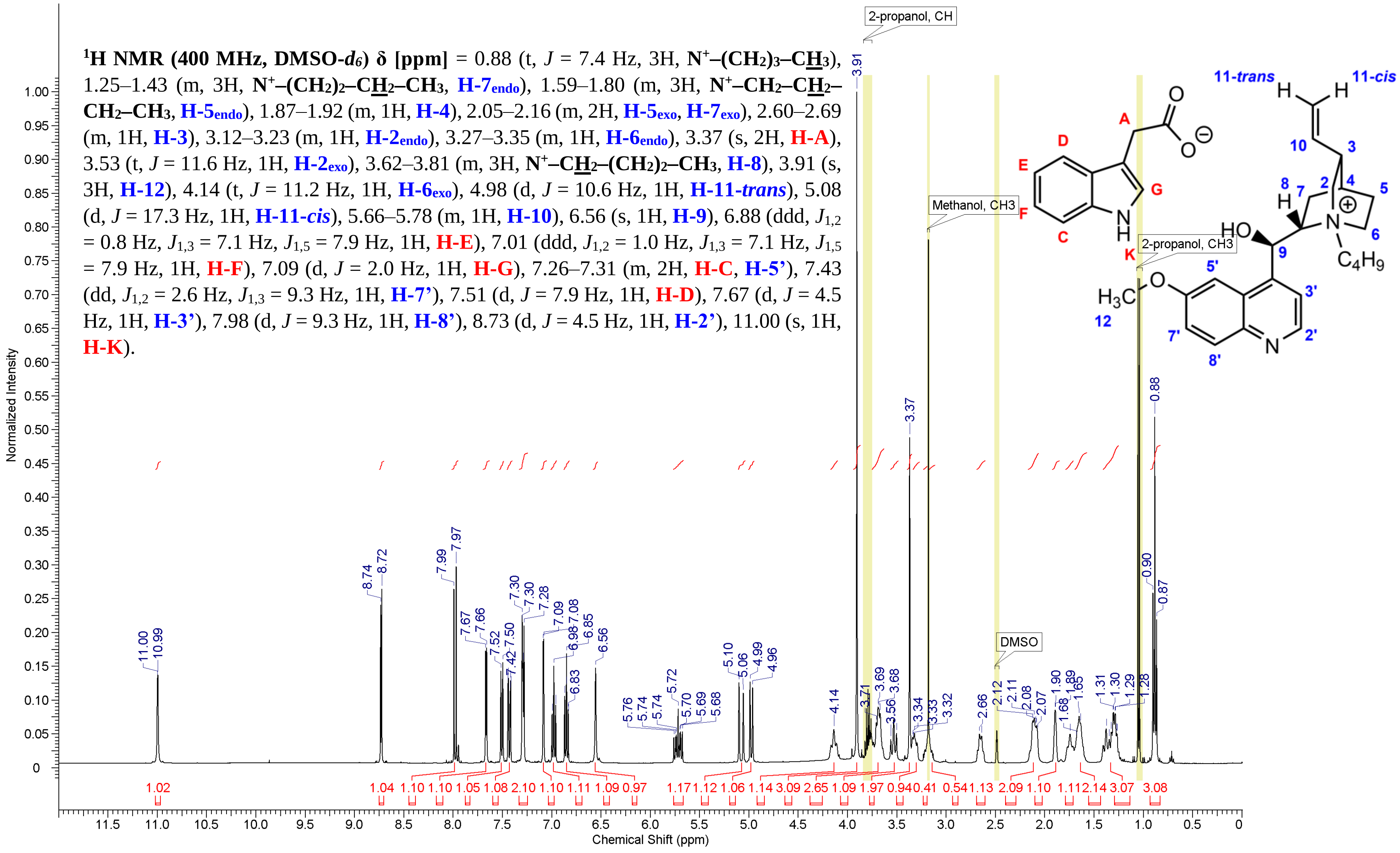

Figure S7. ^{1}H NMR spectrum of 1-butylquininium indole-3-acetate (**2**)

^{13}C NMR (100 MHz, DMSO-*d$_6$*) δ [ppm] = 13.5 (N$^+$–CH$_2$–CH$_2$–CH$_2$–C̲H$_3$), 19.6 (N$^+$–CH$_2$–CH$_2$–C̲H$_2$–CH$_3$), 20.3 (**C-7**), 23.9 (N$^+$–CH$_2$–C̲H$_2$–CH$_2$–CH$_3$), 24.7 (**C-5**), 26.1 (**C-4**), 35.2 (**C-A**), 37.6 (**C-3**), 52.0 (**C-6**), 55.3 (**C-12**), 59.5 (N$^+$–C̲H$_2$–CH$_2$–CH$_2$–CH$_3$, **C-2**), 62.5 (**C-9**), 66.5 (**C-8**), 101.9 (**C-5'**), 111.0 (**C-B**), 111.9 (**C-C**), 116.4 (**C-11**), 117.6 (**C-D**), 119.1 (**C-E**), 120.3 (**C-F**), 120.4 (**C-3'**), 121.3 (**C-7'**), 123.0 (**C-G**), 125.8 (**C-9'**), 127.9 (**C-H**), 131.4 (**C-8'**), 136.2 (**C-I**), 138.1 (**C-10**), 143.8 (**C-10'**), 145.7 (**C-4'**), 147.5 (**C-2'**), 157.2 (**C-6'**), 175.4 (**C-J**).

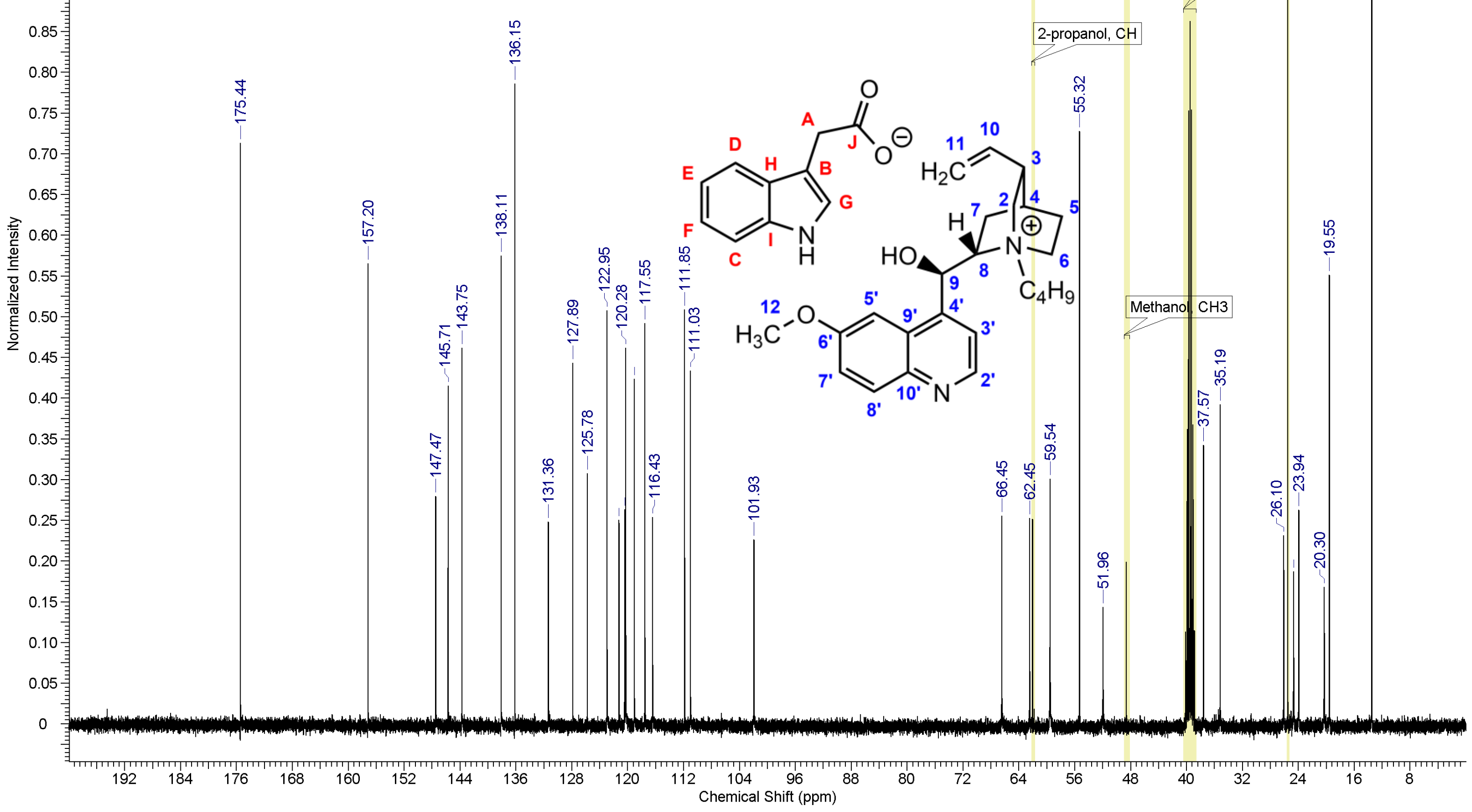

Figure S8. ^{13}C NMR spectrum of 1-butylquininium indole-3-acetate (**2**)

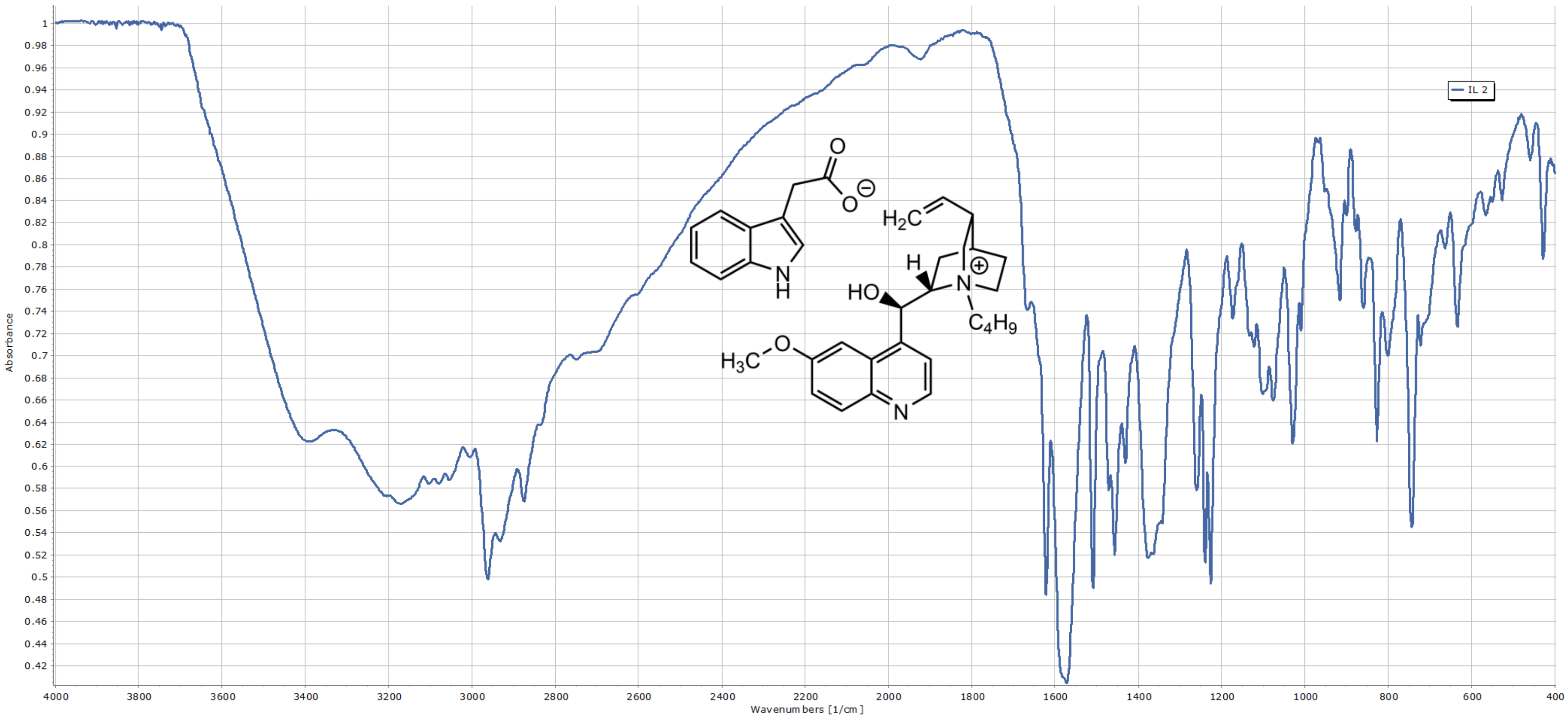

IR (KBr disc) ν [cm^{-1}] = 3386, 3204, 3171, 3103, 3078, 3054, 3004, 2961, 2932, 2875, 2749, 1621, 1572, 1508, 1457, 1431, 1376, 1259, 1239, 1226, 1173, 1122, 1101, 1076, 1029, 916, 859, 826, 800, 743, 663, 634, 565, 527, 459, 427.

Figure S9. FT-IR spectrum of 1-butylquininium indole-3-acetate (**2**)

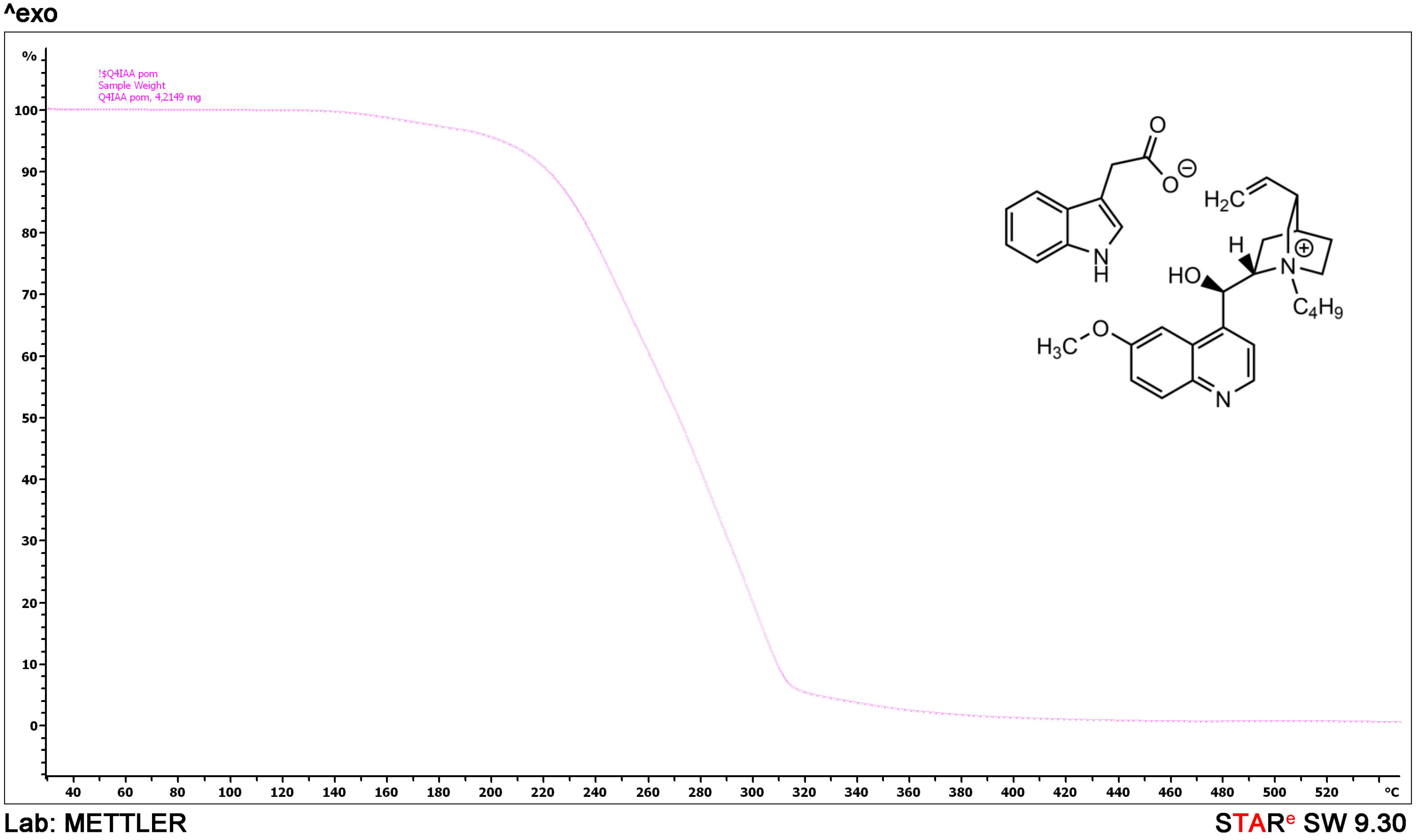

Figure S10. TGA plot of 1-butylquininium indole-3-acetate (**2**)

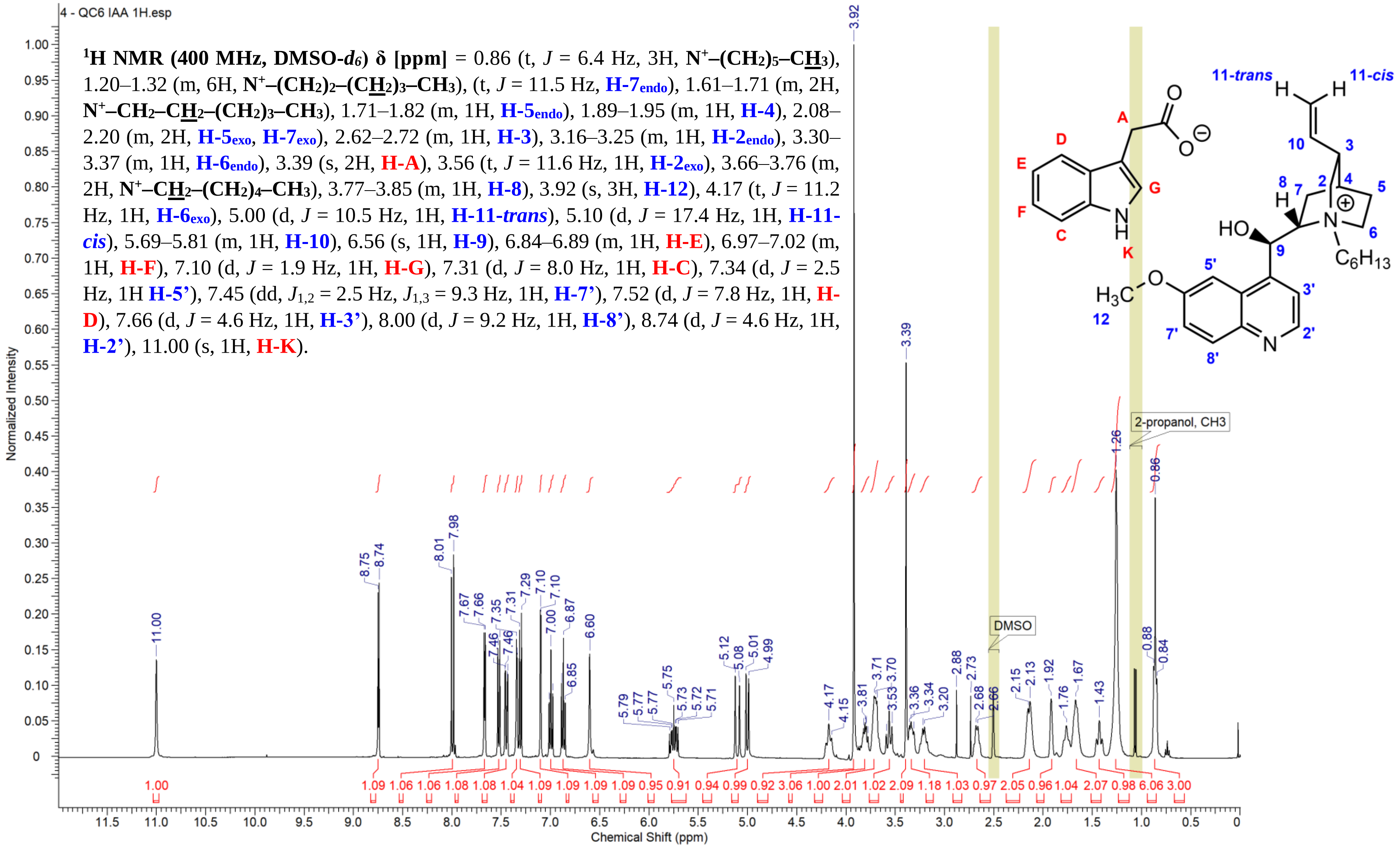

Figure S11. ^{1}H NMR spectrum of 1-hexylquininium indole-3-acetate (**3**)

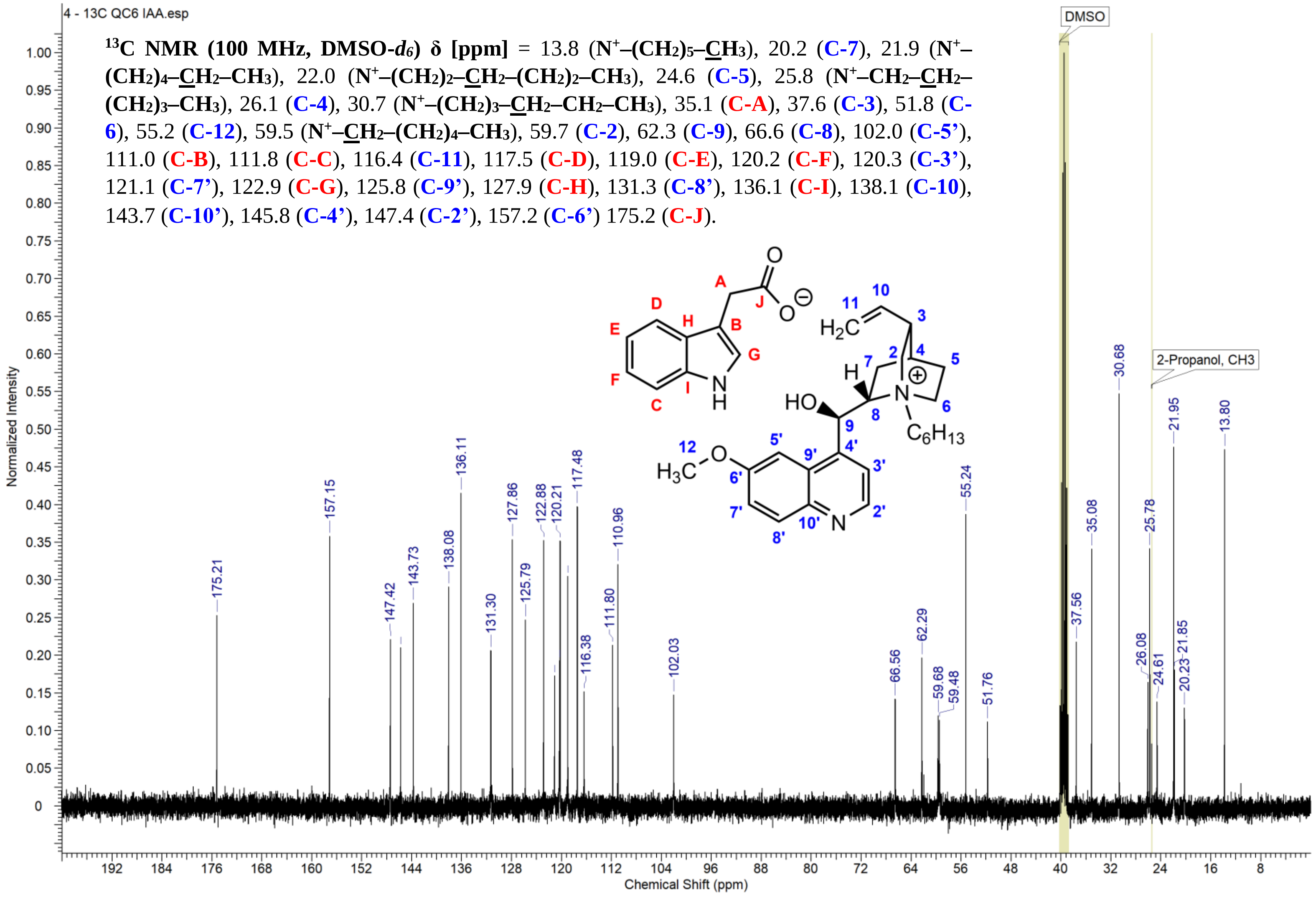

Figure S12. ^{13}C NMR spectrum of 1-hexylquininium indole-3-acetate (**3**)

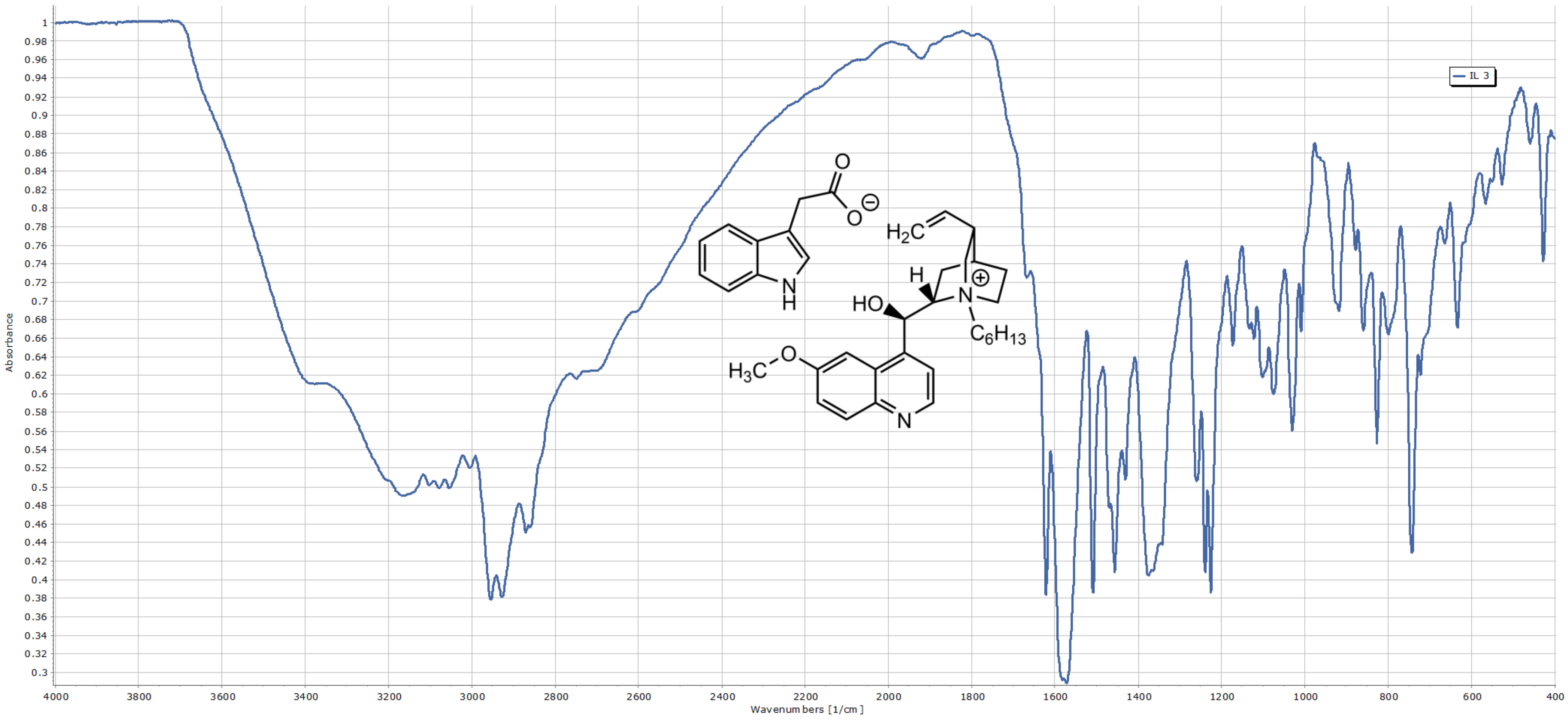

IR (KBr disc) ν [cm^{-1}] = 3377, 3165, 3102, 3078, 3054, 3004, 2954, 2928, 2870, 2749, 2701, 1621, 1572, 1508, 1457, 1431, 1375, 1259, 1239, 1226, 1173, 1123, 1102, 1075, 1030, 1009, 918, 878, 859, 826, 799, 743, 663, 634, 566, 527, 459, 427.

Figure S13. FT-IR spectrum of 1-hexylquininium indole-3-acetate (**3**)

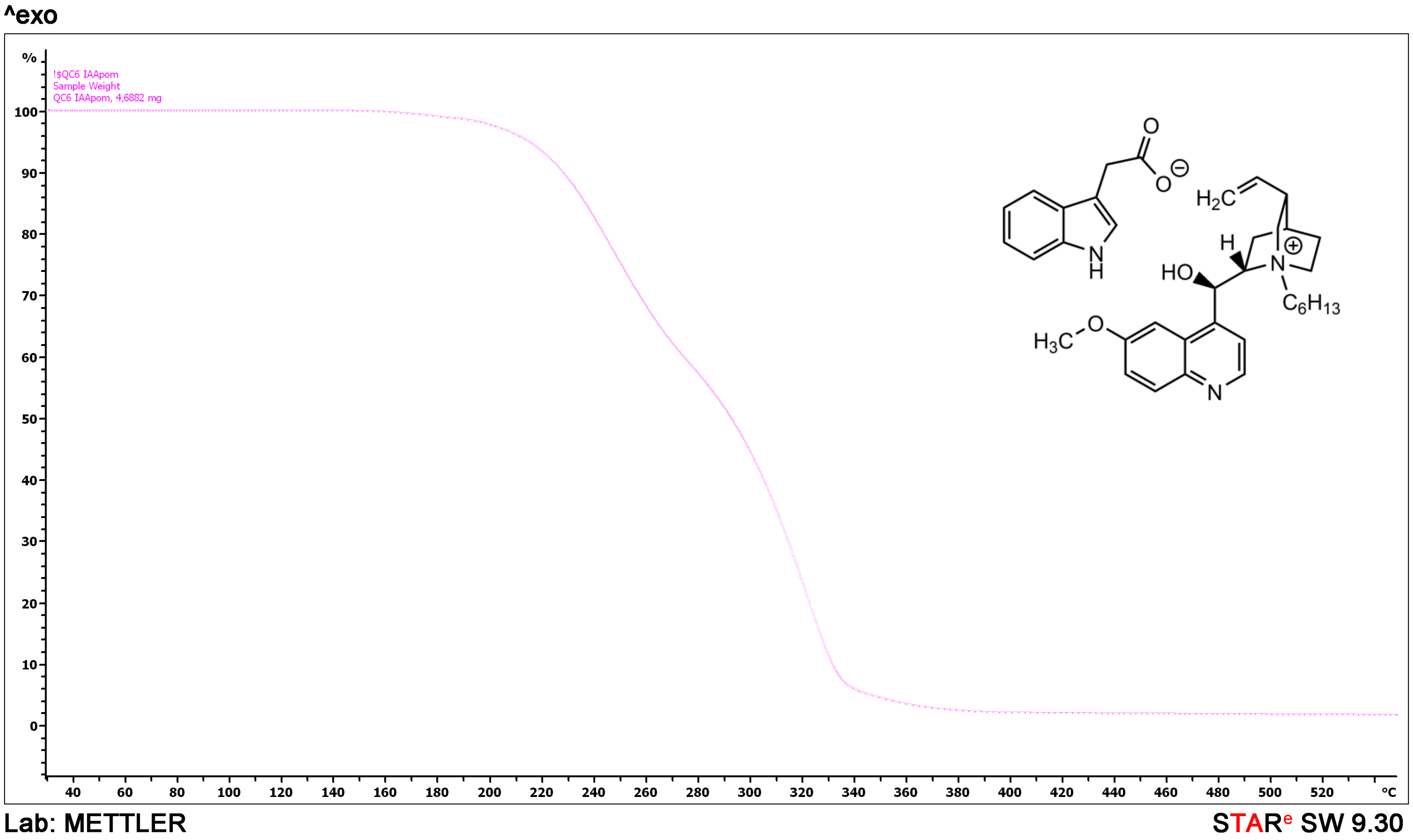

Figure S14. TGA plot of 1-hexylquininium indole-3-acetate (**3**)

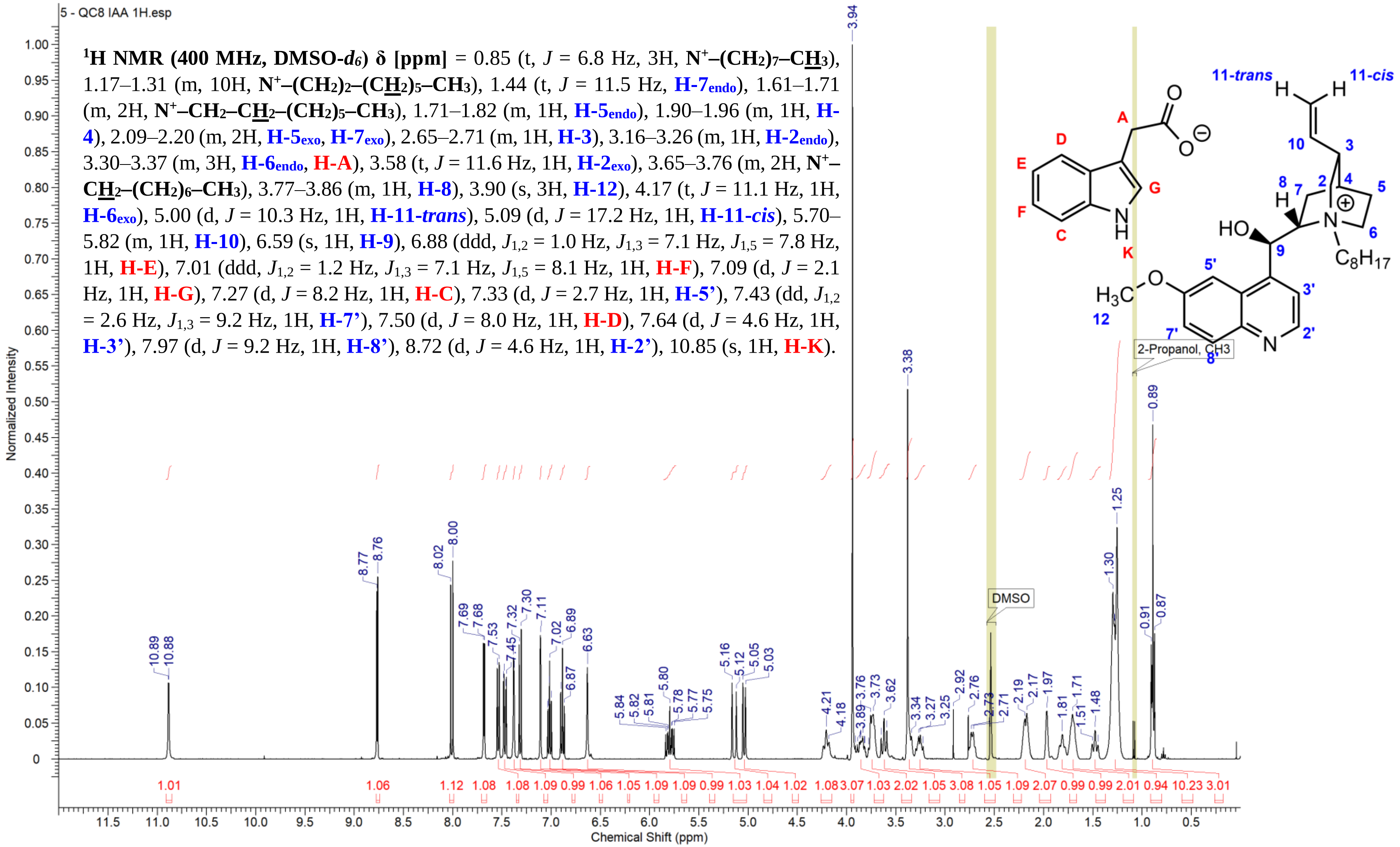

Figure S15. ^{1}H NMR spectrum of 1-octylquininium indole-3-acetate (**4**)

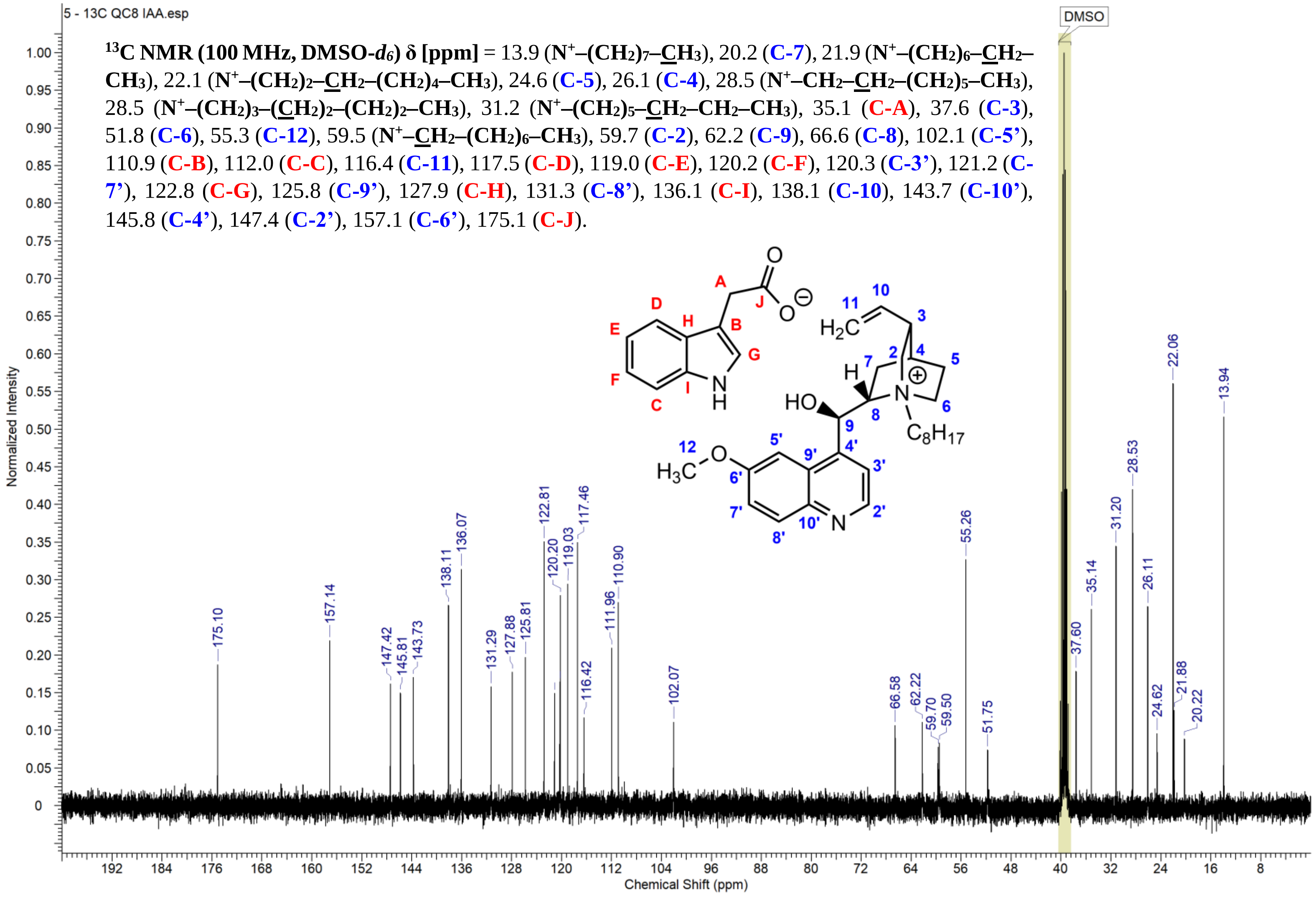

Figure S16. ^{13}C NMR spectrum of 1-octylquininium indole-3-acetate (**4**)

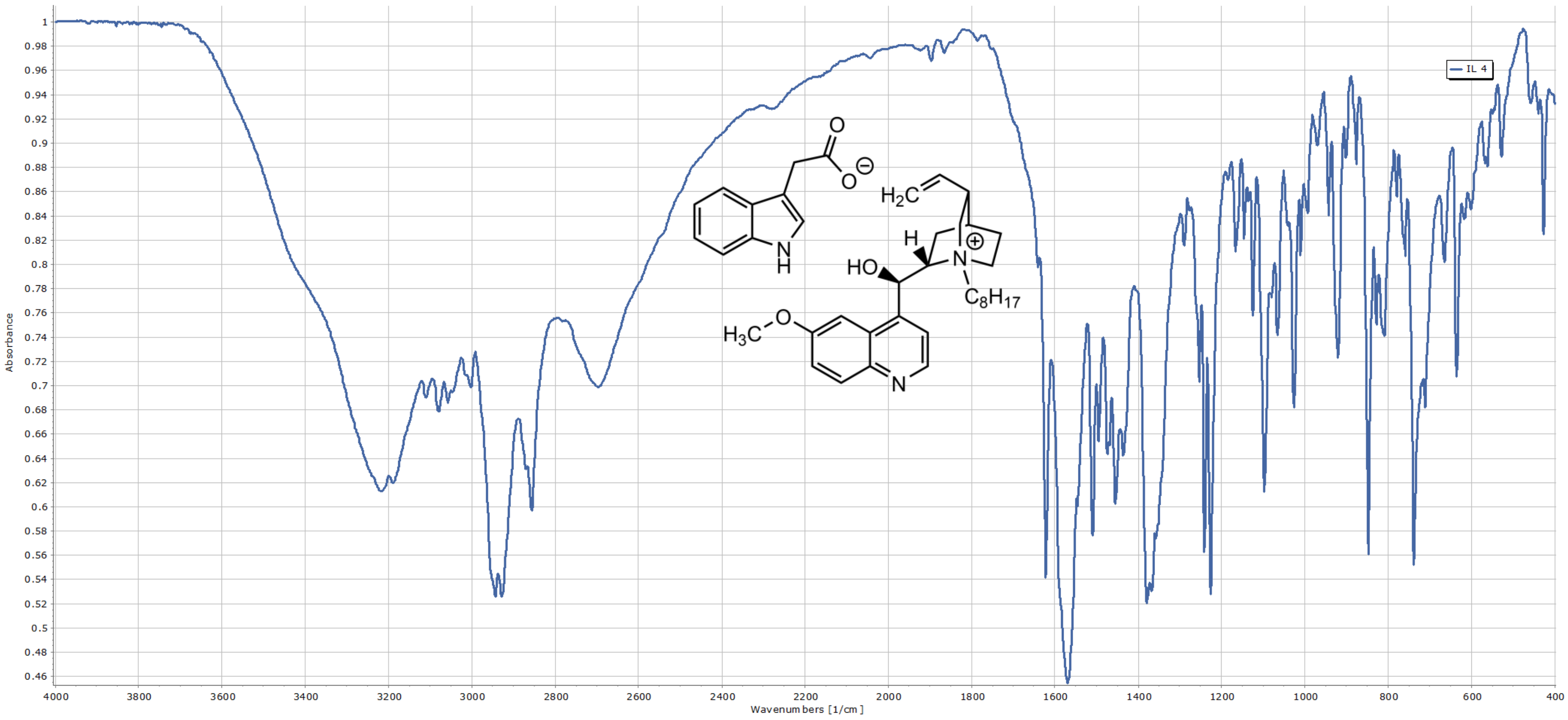

IR (KBr disc) ν [cm^{-1}] = 3218, 3189, 3110, 3079, 3058, 3002, 2943, 2928, 2856, 2782, 2696, 1622, 1569, 1509, 1495, 1474, 1455, 1436, 1379, 1289, 1241, 1227, 1184, 1166, 1146, 1124, 1097, 1067, 1026, 993, 969, 943, 920, 877, 848, 828, 809, 779, 759, 738, 712, 664, 636, 617, 601, 561, 527, 457, 426.

Figure S17. FT-IR spectrum of 1-octylquininium indole-3-acetate (**4**)

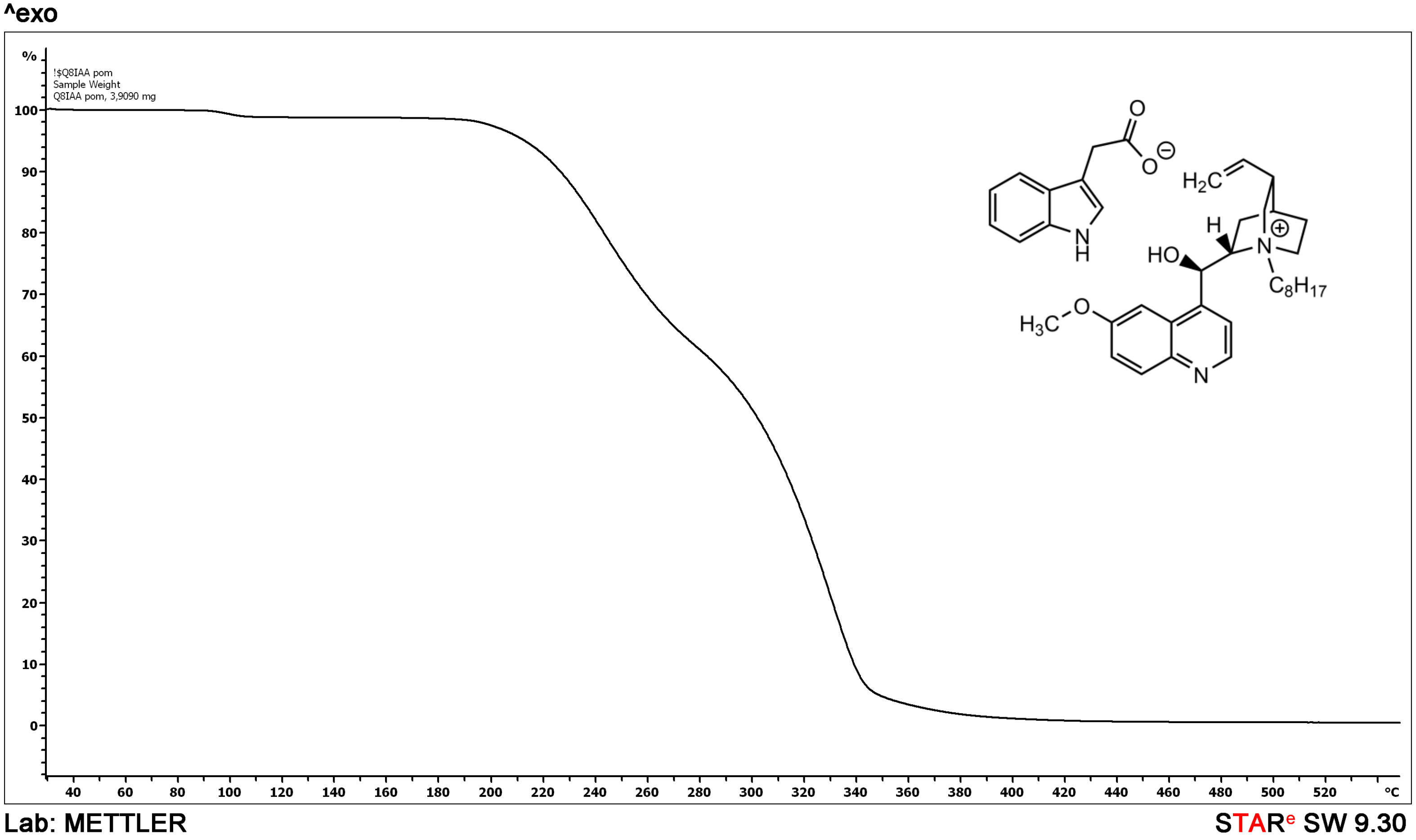

Figure S18. TGA plot of 1-octylquininium indole-3-acetate (**4**)

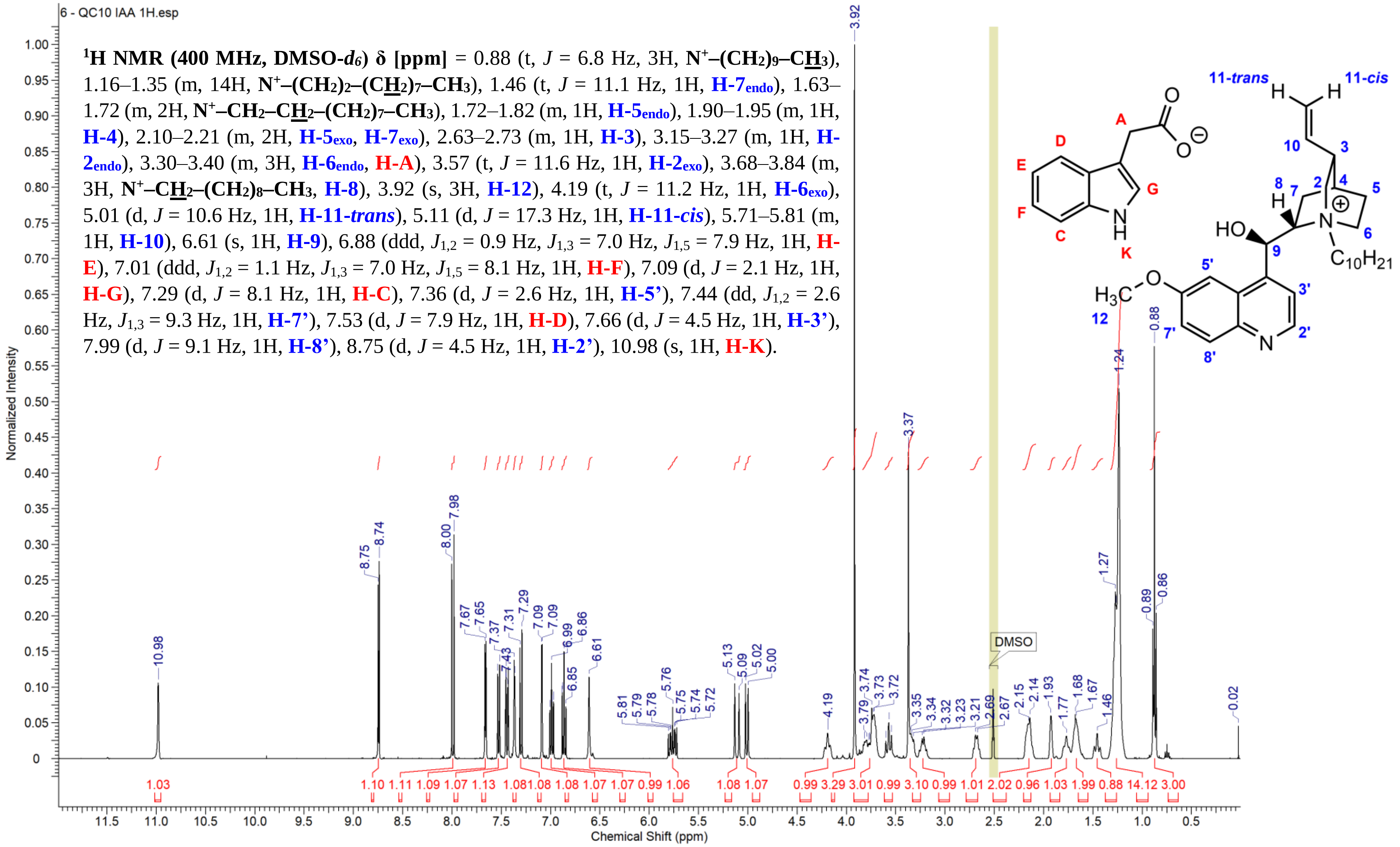

Figure S19. ^{1}H NMR spectrum of 1-decylquininium indole-3-acetate (**5**)

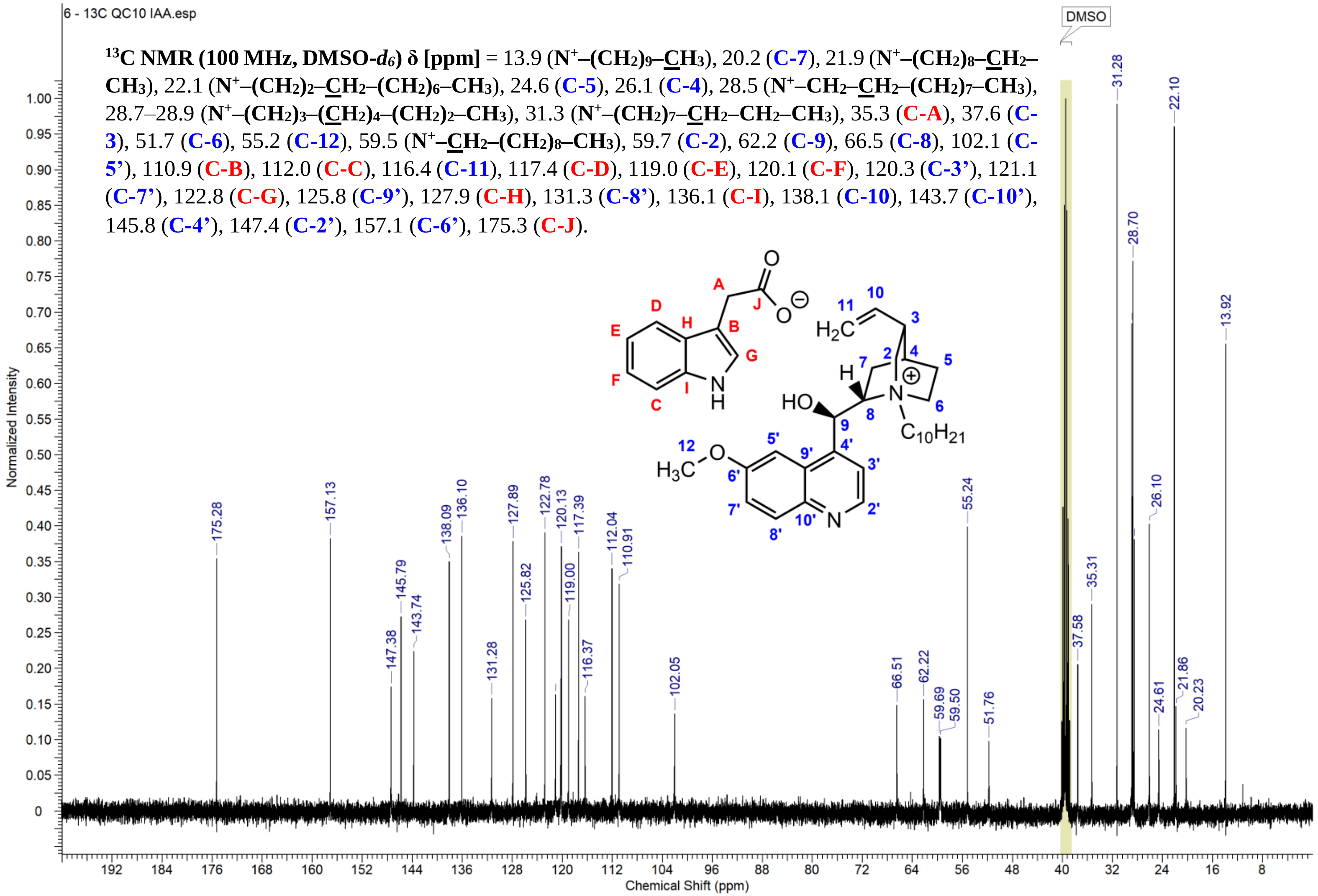

Figure S20. ^{13}C NMR spectrum of 1-decylquininium indole-3-acetate (**5**)

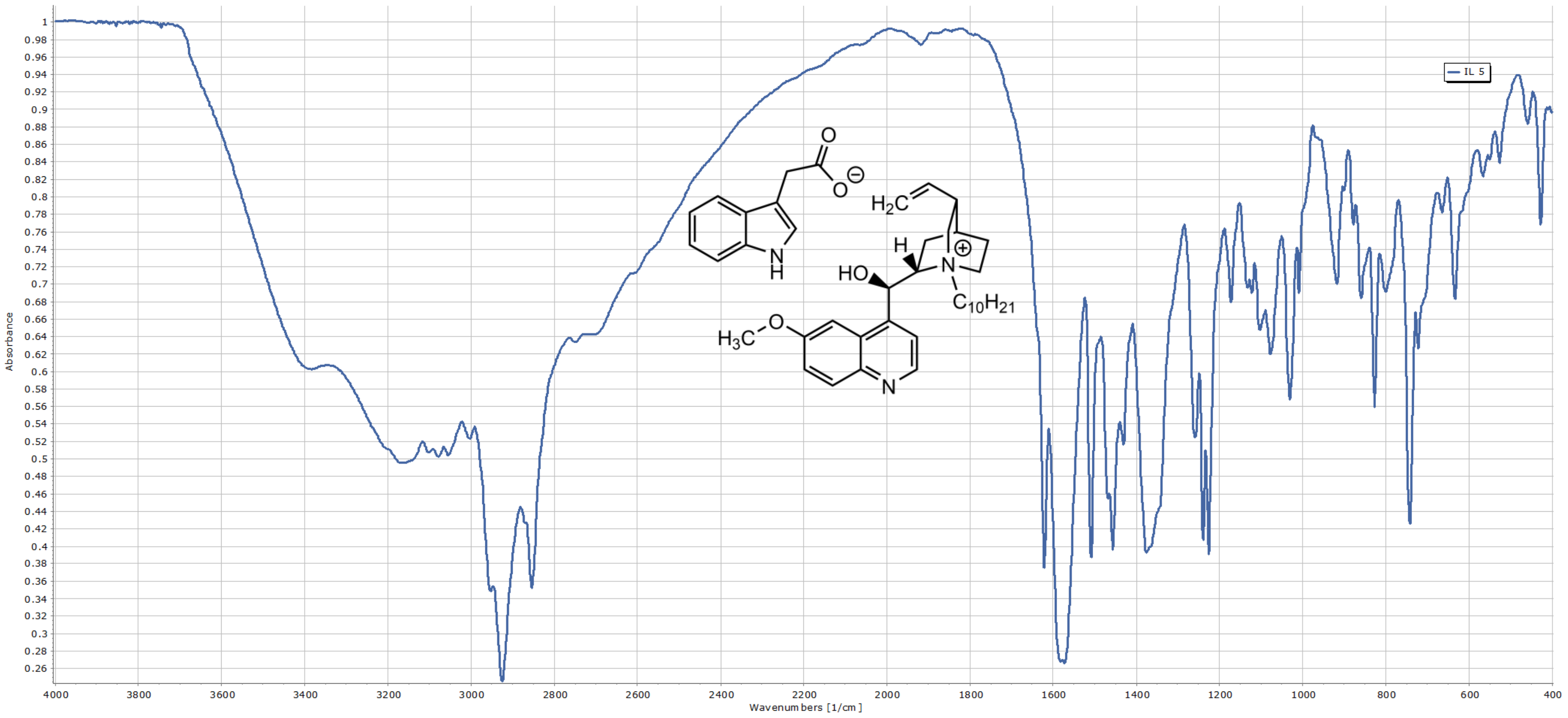

IR (KBr disc) ν [cm^{-1}] = 3384, 3163, 3102, 3078, 3055, 3003, 2953, 2925, 2853, 2748, 1621, 1572, 1508, 1457, 1431, 1375, 1258, 1239, 1226, 1173, 1123, 1103, 1077, 1031, 1009, 918, 878, 859, 826, 800, 742, 664, 634, 566, 527, 458, 427.

Figure S21. FT-IR spectrum of 1-decylquininium indole-3-acetate (**5**)

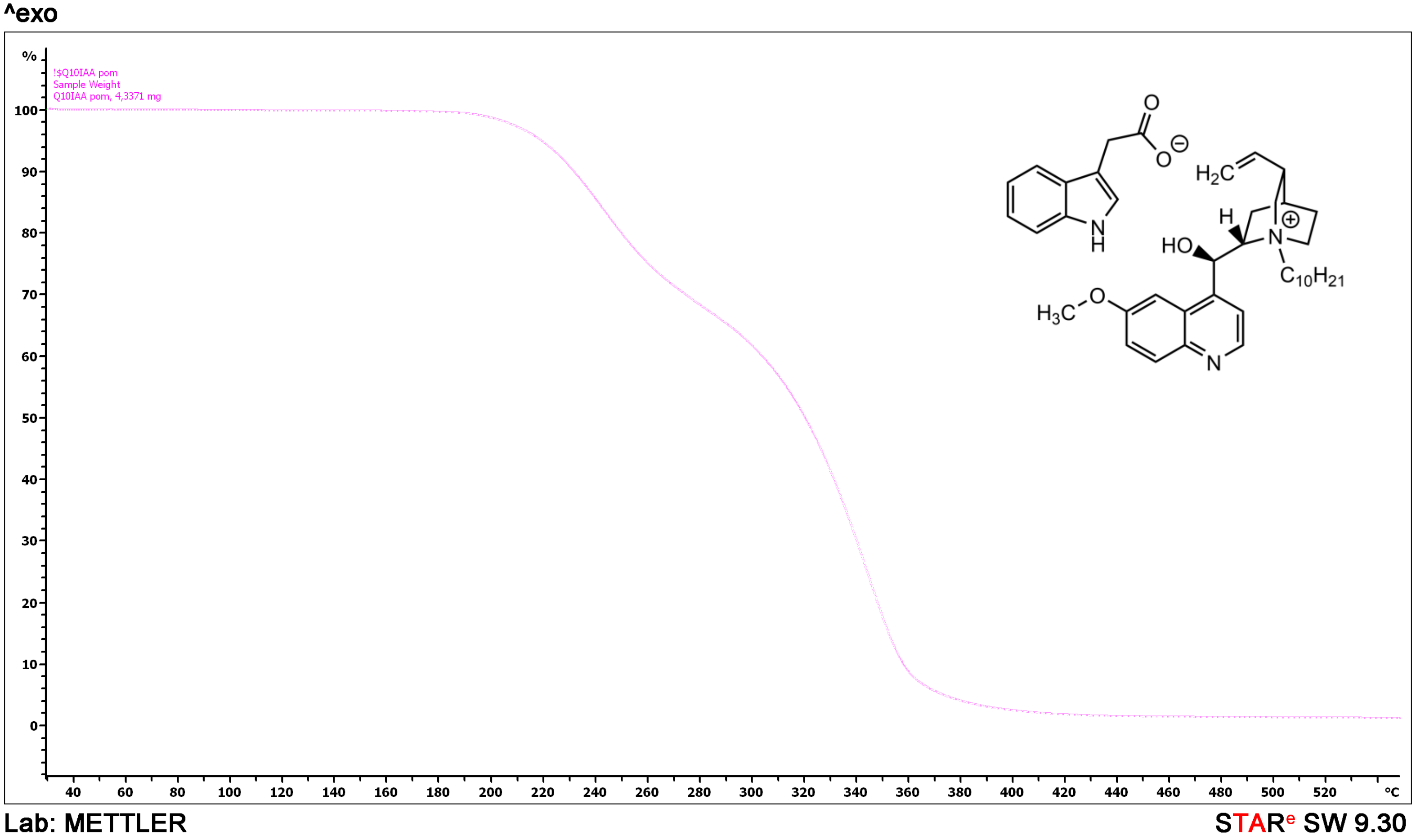

Figure S22. TGA plot of 1-decylquininium indole-3-acetate (**5**)

^{1}H NMR (400 MHz, DMSO-d_6) δ [ppm] = 0.85 (t, J = 6.8 Hz, 3H, **N$^+$–(CH$_2$)$_{11}$–C<u>H</u>$_3$**), 1.16–1.31 (m, 18H, **N$^+$–(CH$_2$)$_2$–(C<u>H</u>$_2$)$_9$–CH$_3$**), 1.43 (t, J = 11.5 Hz, 1H, **H-7$_{endo}$**), 1.58–1.68 (m, 2H, **N$^+$–CH$_2$–C<u>H</u>$_2$–(CH$_2$)$_9$–CH$_3$**), 1.69–1.78 (m, 1H, **H-5$_{endo}$**), 1.86–1.91 (m, 1H, **H-4**), 2.05–2.20 (m, 2H, **H-5$_{exo}$**, **H-7$_{exo}$**), 2.60–2.69 (m, 1H, **H-3**), 3.14–3.24 (m, 1H, **H-2$_{endo}$**), 3.25–3.33 (m, 1H, **H-6$_{endo}$**), 3.35 (s, 2H, **H-A**), 3.53 (t, J = 11.5 Hz, 1H, **H-2$_{exo}$**), 3.63–3.74 (m, 3H, **N$^+$–C<u>H</u>$_2$–(CH$_2$)$_{10}$–CH$_3$**, **H-8**), 3.89 (s, 3H, **H-12**), 4.16 (t, J = 10.5 Hz, 1H, **H-6$_{exo}$**), 4.98 (d, J = 10.4 Hz, 1H, **H-11-*trans***), 5.08 (d, J = 17.3 Hz, 1H, **H-11-*cis***), 5.67–5.79 (m, 1H, **H-10**), 6.58 (s, 1H, **H-9**), 6.88 (ddd, $J_{1,2}$ = 0.8 Hz, $J_{1,3}$ = 7.1 Hz, $J_{1,5}$ = 7.9 Hz, 1H, **H-E**), 7.01 (ddd, $J_{1,2}$ = 1.0 Hz, $J_{1,3}$ = 7.0 Hz, $J_{1,5}$ = 8.0 Hz, 1H, **H-F**), 7.06 (d, J = 2.0 Hz, 1H, **H-G**), 7.29 (d, J = 8.0 Hz, 1H, **H-C**), 7.34 (d, J = 2.5 Hz, 1H, **H-5’**), 7.41 (dd, $J_{1,2}$ = 2.6 Hz, $J_{1,3}$ = 9.2 Hz, 1H, **H-7’**), 7.50 (d, J = 7.8 Hz, 1H, **H-D**), 7.63 (d, J = 4.6 Hz, 1H, **H-3’**), 7.99 (d, J = 9.3 Hz, 1H, **H-8’**), 8.71 (d, J = 4.5 Hz, 1H, **H-2’**), 10.98 (s, 1H, **H-K**).

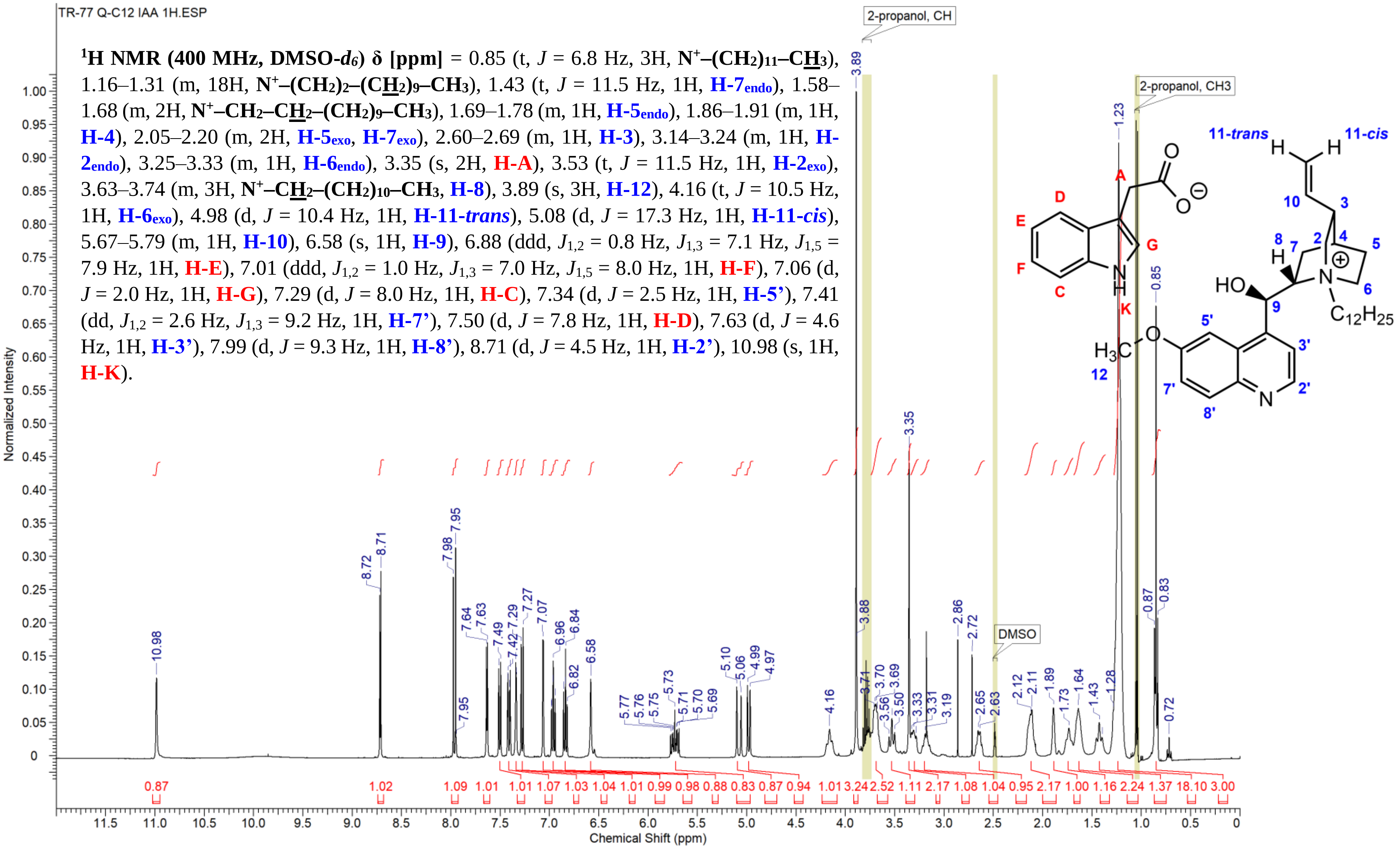

Figure S23. ^{1}H NMR spectrum of 1-dodecylquininium indole-3-acetate (**6**)

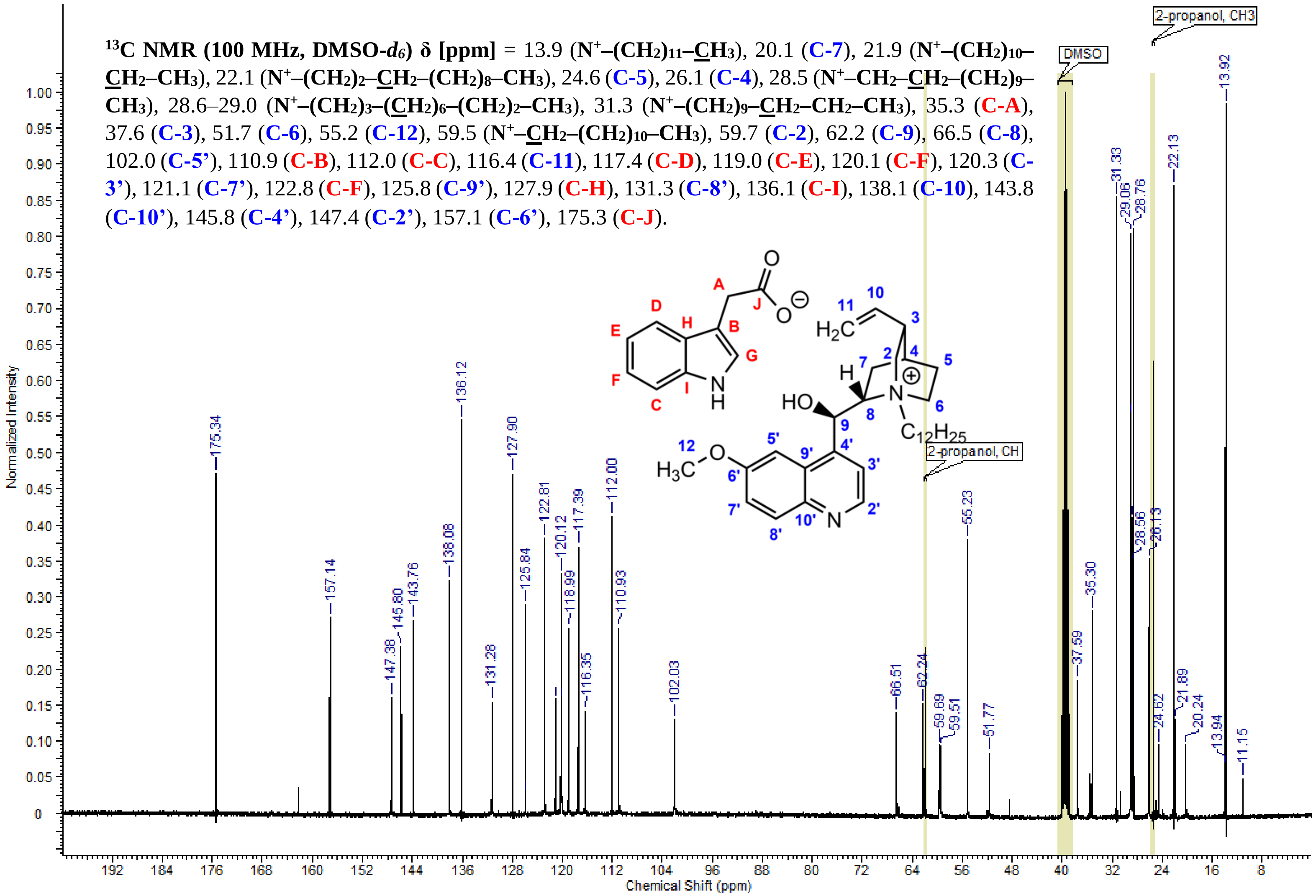

Figure S24. ^{13}C NMR spectrum of 1-dodecylquininium indole-3-acetate (**6**)

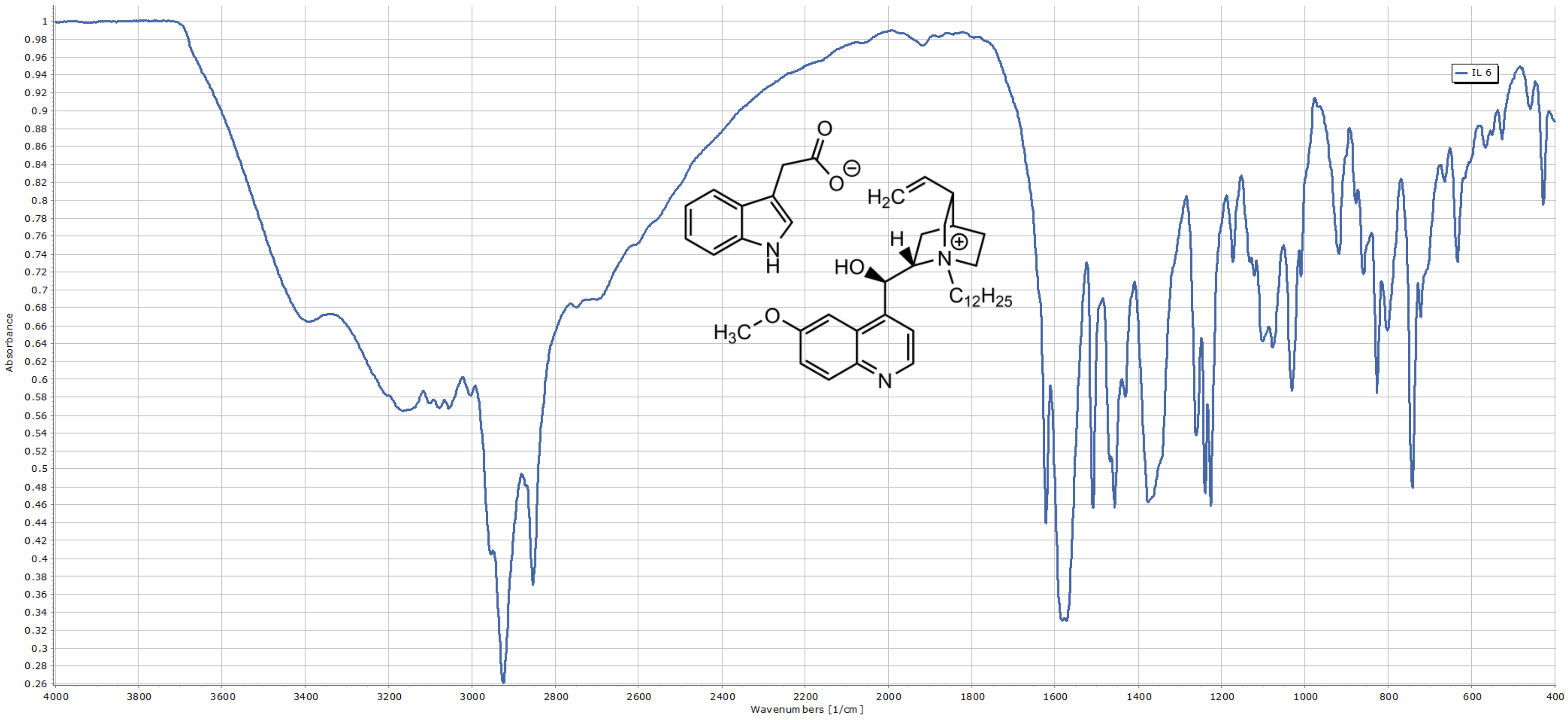

IR (KBr disc) ν [cm^{-1}] = 3391, 3166, 3102, 3078, 3055, 3003, 2954, 2925, 2853, 2750, 2700, 1621, 1572, 1508, 1457, 1431, 1376, 1260, 1239, 1226, 1173, 1101, 1077, 1031, 1009, 918, 878, 859, 826, 801, 742, 664, 634, 567, 527, 458, 427.

Figure S25. FT-IR spectrum of 1-dodecylquininium indole-3-acetate (**6**)

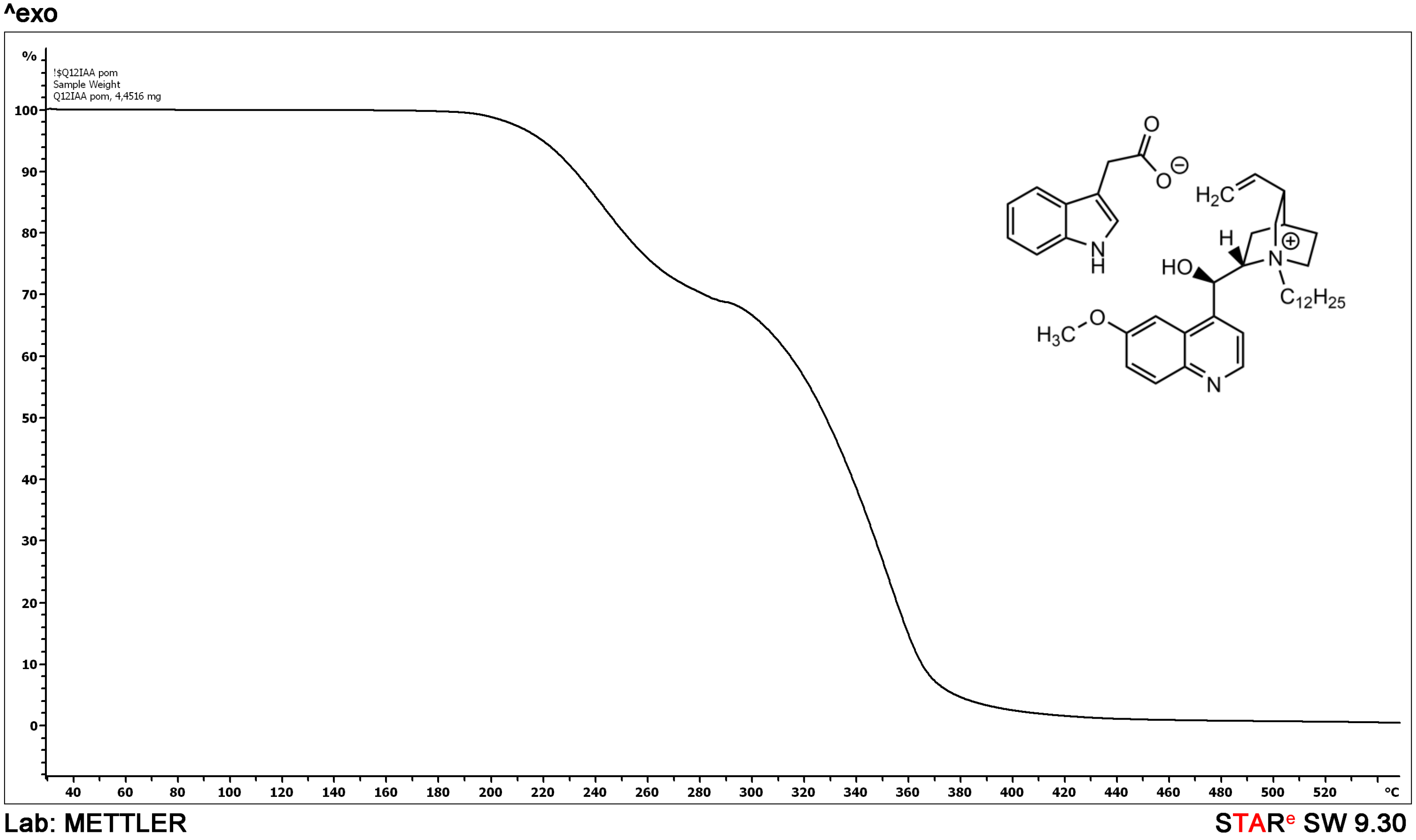

Figure S26. TGA plot of 1-dodecylquininium indole-3-acetate (**6**)

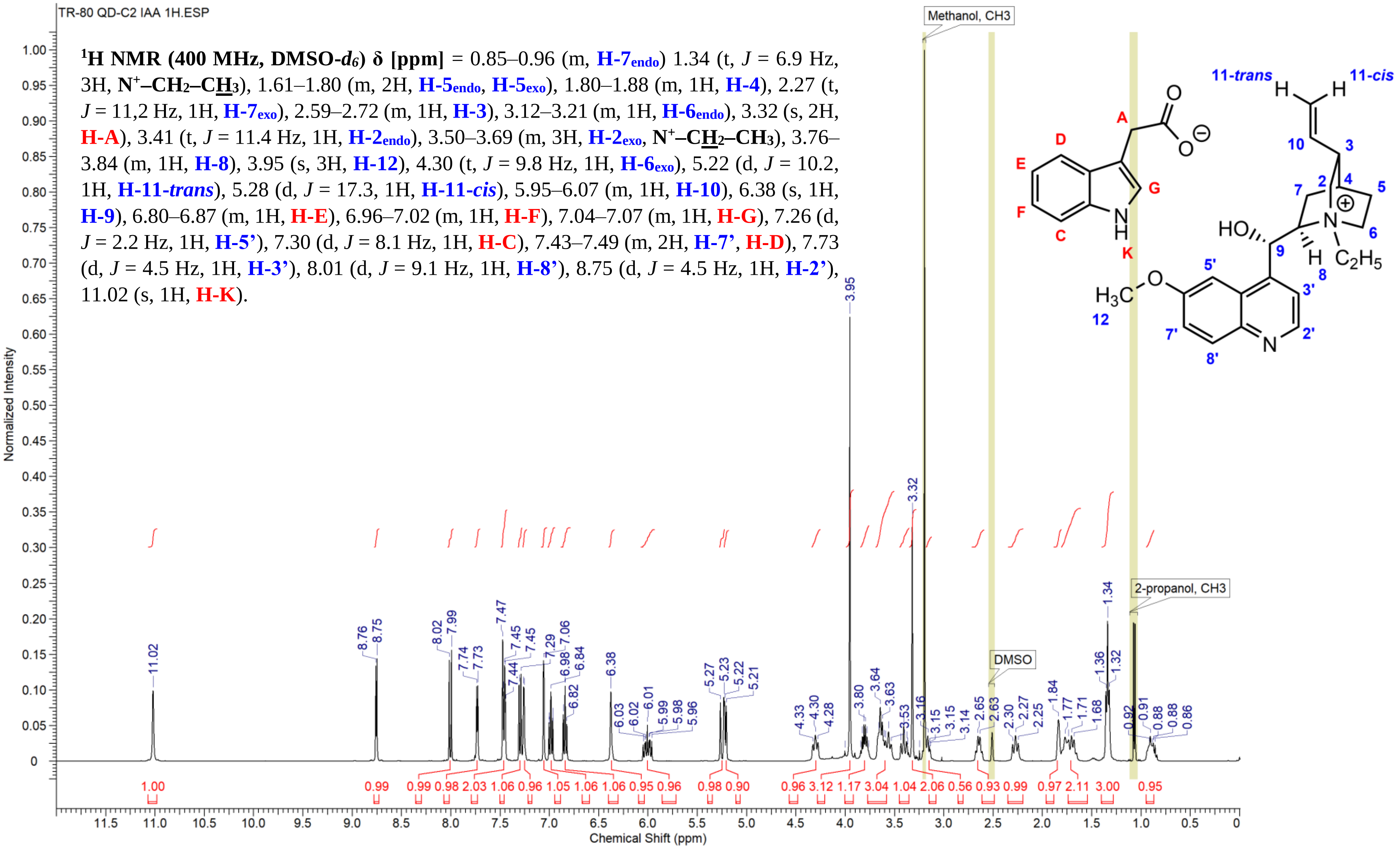

Figure S27. ^{1}H NMR spectrum of 1-ethylquinidinium indole-3-acetate (**7**)

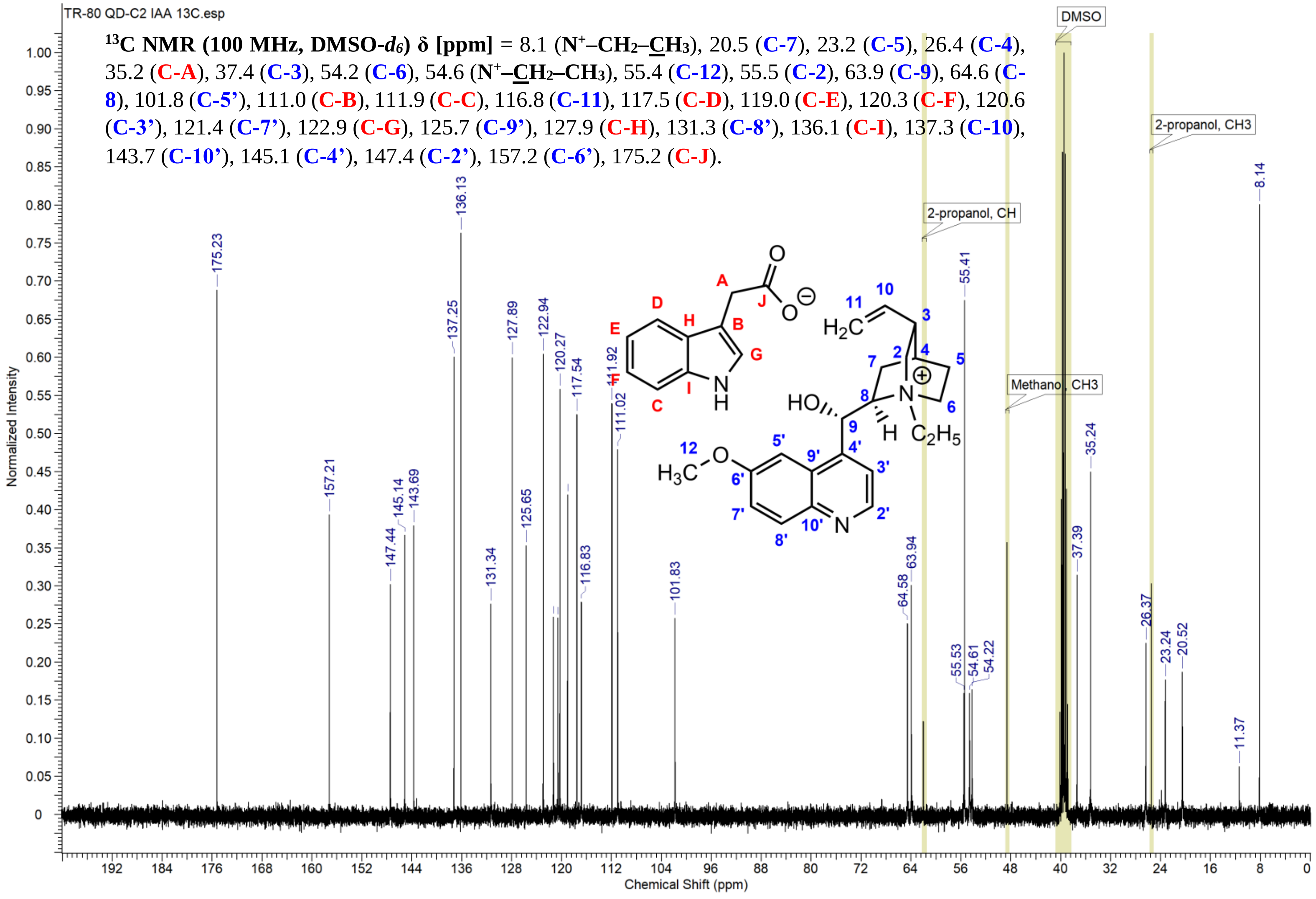

Figure S28. ^{13}C NMR spectrum of 1-ethylquinidinium indole-3-acetate (**7**)

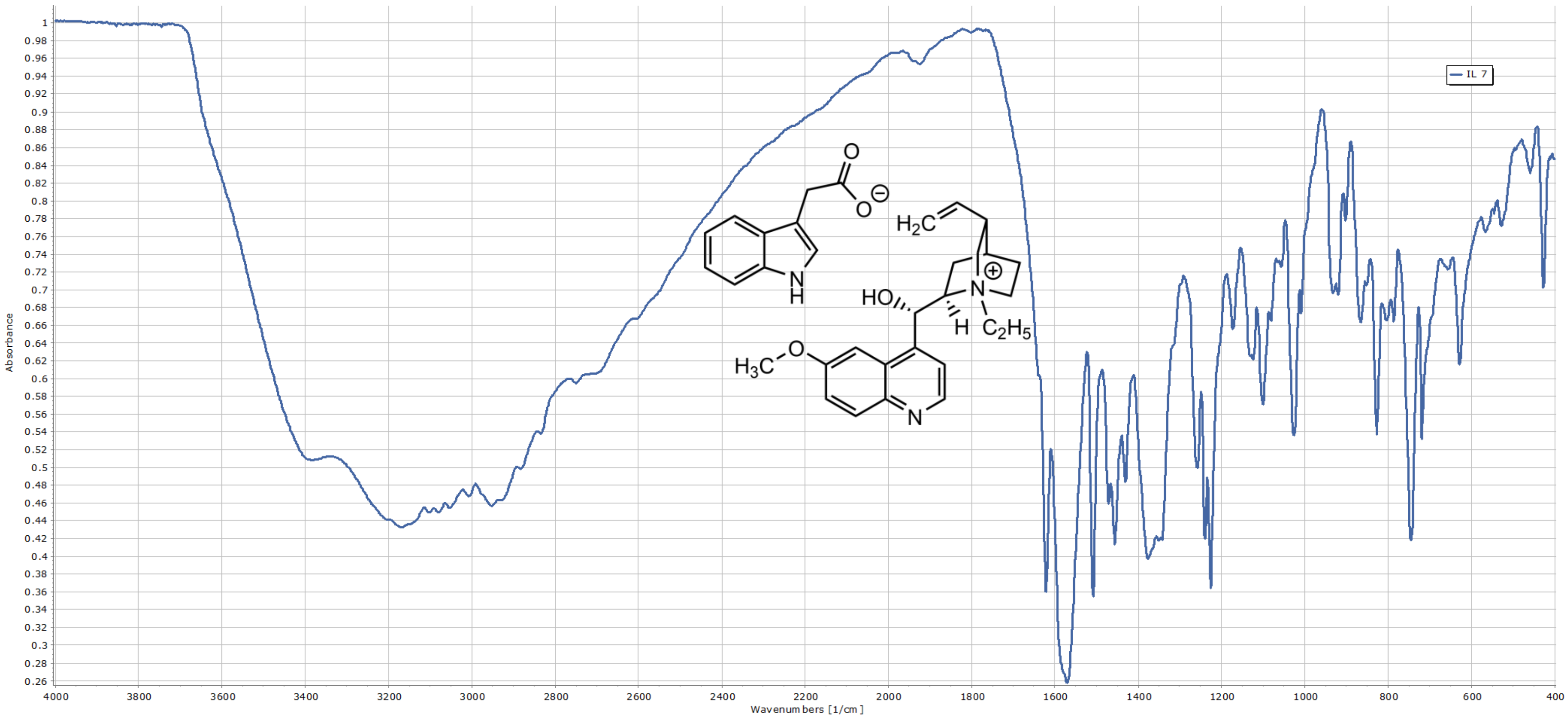

IR (KBr disc) ν [cm⁻¹] = 3383, 3170, 3103, 3079, 3054, 3008, 2952, 2882, 2834, 2750, 1621, 1570, 1508, 1456, 1431, 1376, 1350, 1258, 1240, 1226, 1173, 1124, 1100, 1056, 1026, 932, 920, 902, 866, 827, 803, 786, 744, 718, 657, 629, 566, 528, 459, 427.

Figure S29. FT-IR spectrum of 1-ethylquinidinium indole-3-acetate (**7**)

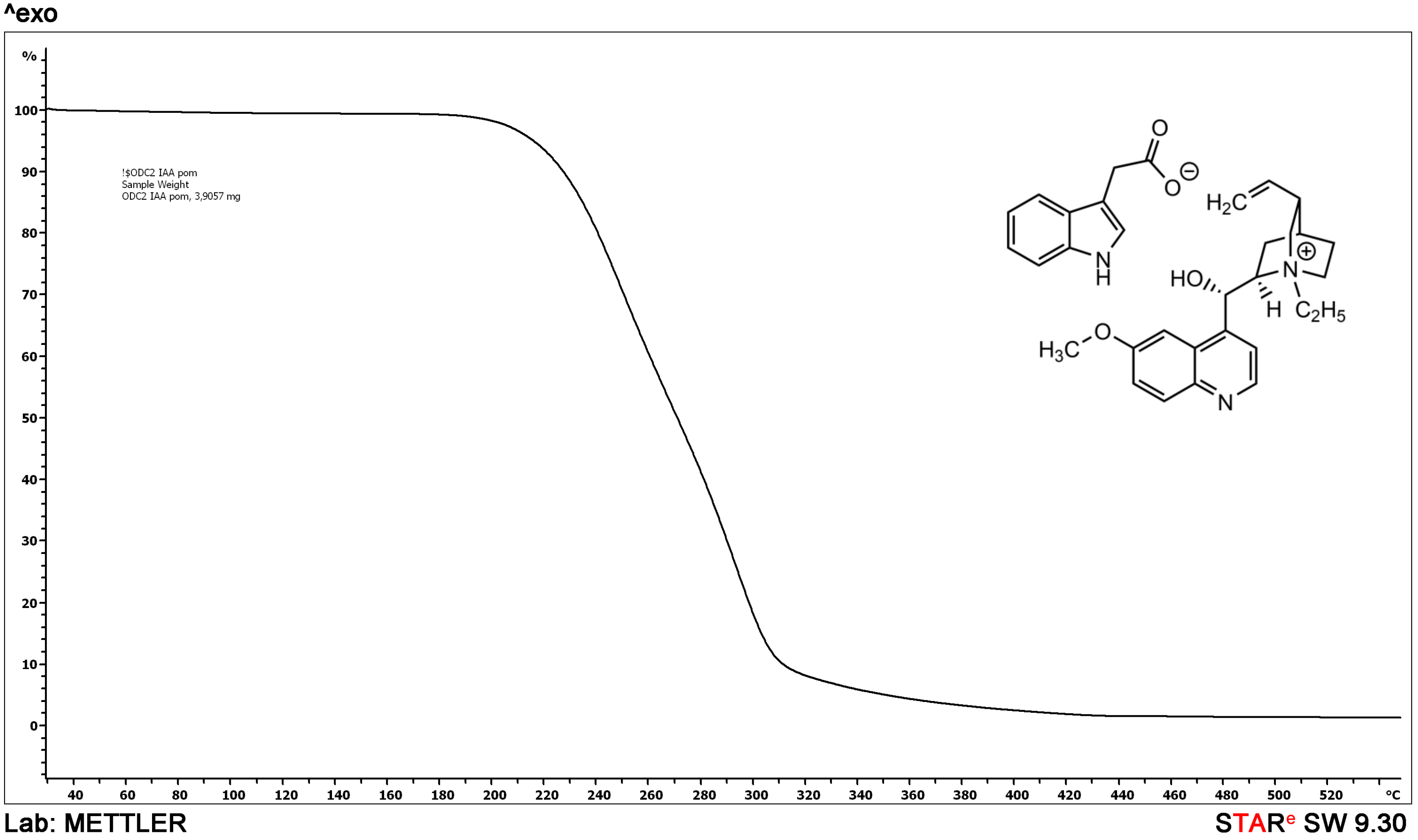

Figure S30. TGA plot of 1-ethylquinidinium indole-3-acetate (**7**)

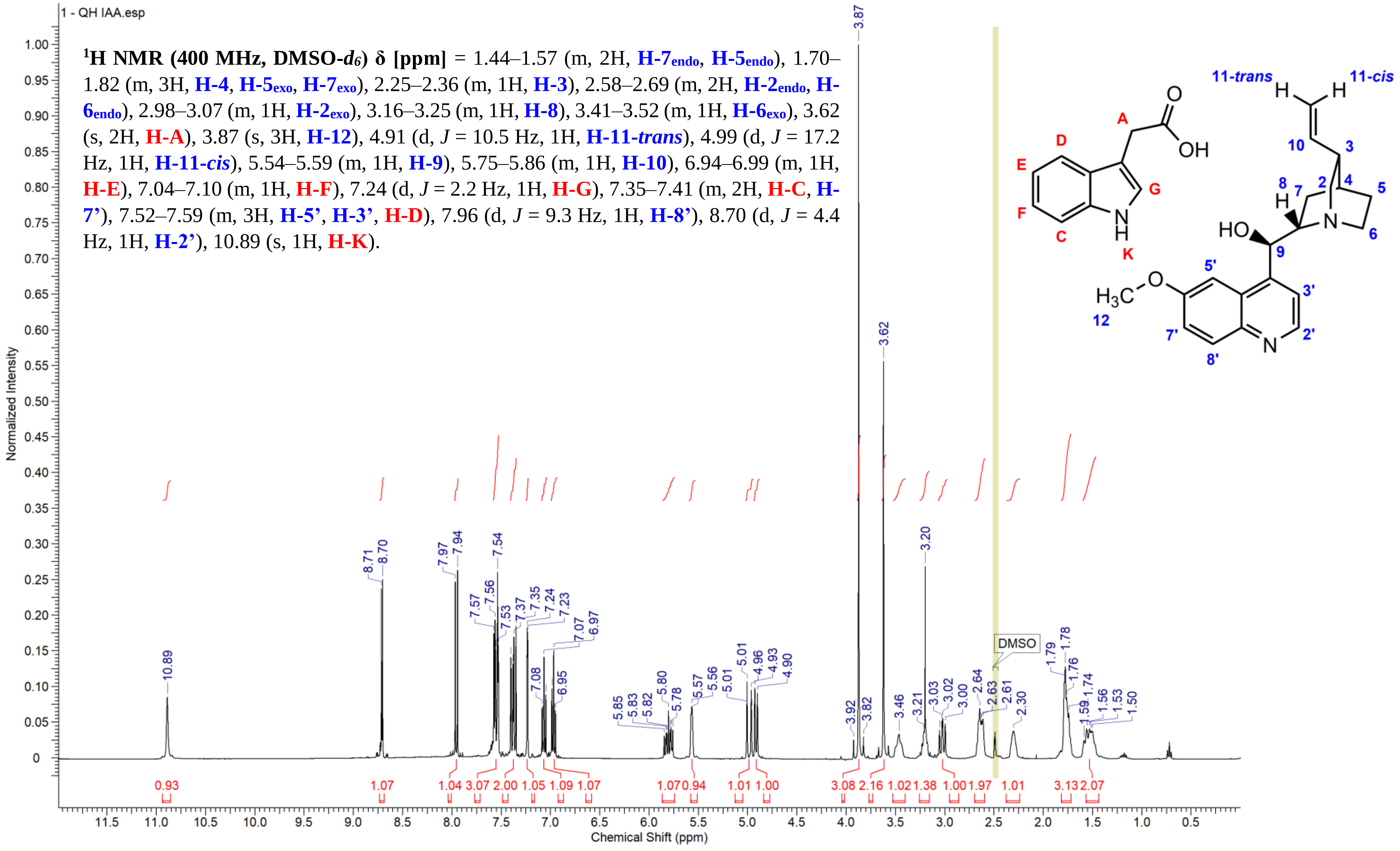

Figure S31. ^{1}H NMR spectrum of binary mixture of quinine and indole-3-acetic acid (IAA+Q)

^{13}C NMR (100 MHz, DMSO-d_6) δ [ppm] = 22.0 (**C-7**), 26.4 (**C-5**), 27.3 (**C-4**), 32.2 (**C-A**), 38.7 (**C-3**), 41.9 (**C-6**), 54.9 (**C-2**), 55.6 (**C-12**), 59.8 (**C-8**), 69.4 (**C-9**), 102.1 (**C-5'**), 108.9 (**C-B**), 111.3 (**C-C**), 114.5 (**C-11**), 118.2 (**C-D**), 118.7 (**C-E**), 119.1 (**C-3'**), 120.8 (**C-F**), 121.2 (**C-7'**), 123.7 (**C-G**), 126.6 (**C-9'**), 127.4 (**C-H**), 131.2 (**C-8'**), 136.1 (**C-I**), 141.6 (**C-10**), 143.9 (**C-10'**), 147.4 (**C-2'**), 148.2 (**C-4'**), 157.2 (**C-6'**), 174.4 (**C-J**).

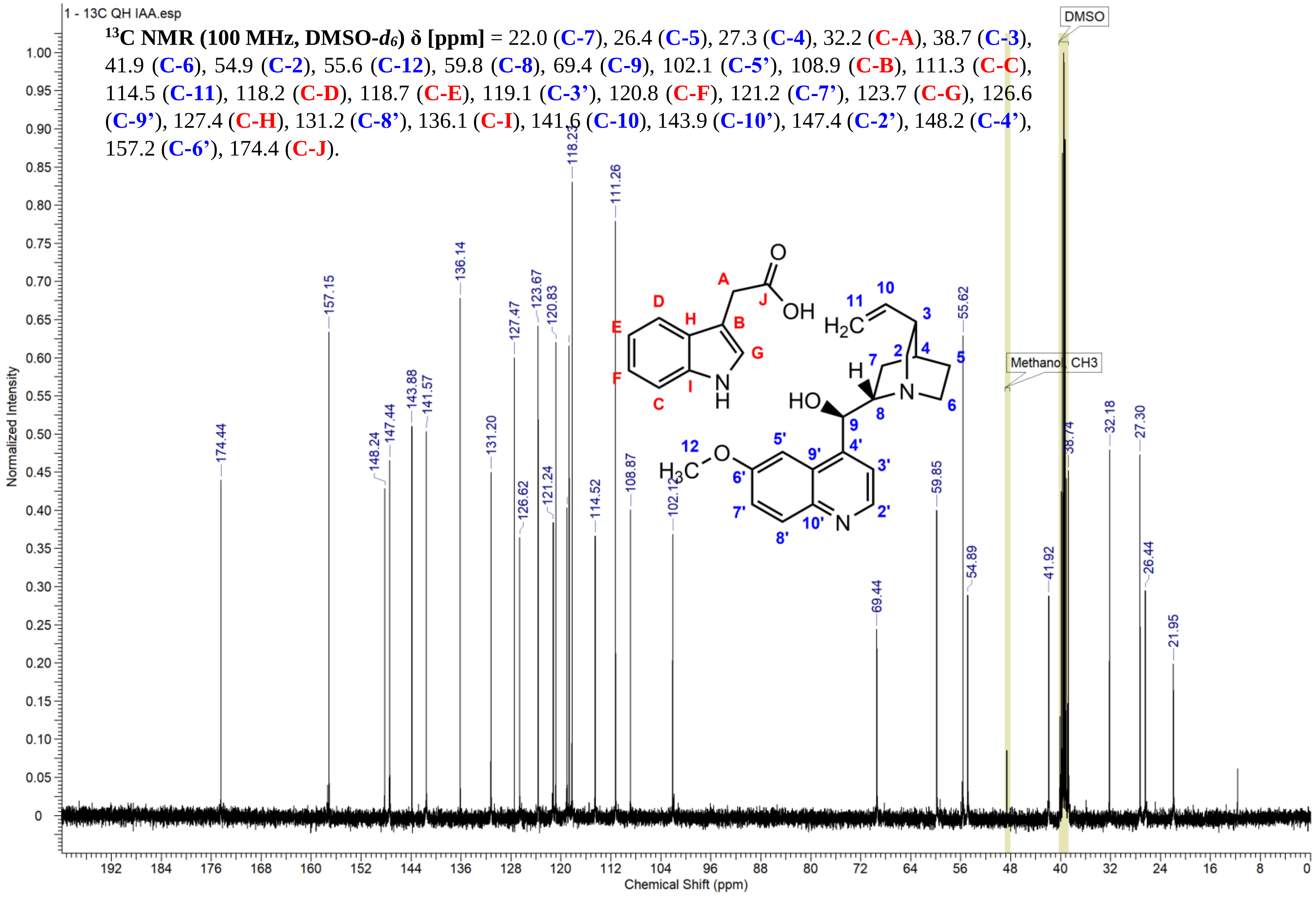

Figure S32. ^{13}C NMR spectrum of binary mixture of quinine and indole-3-acetic acid (IAA+Q)

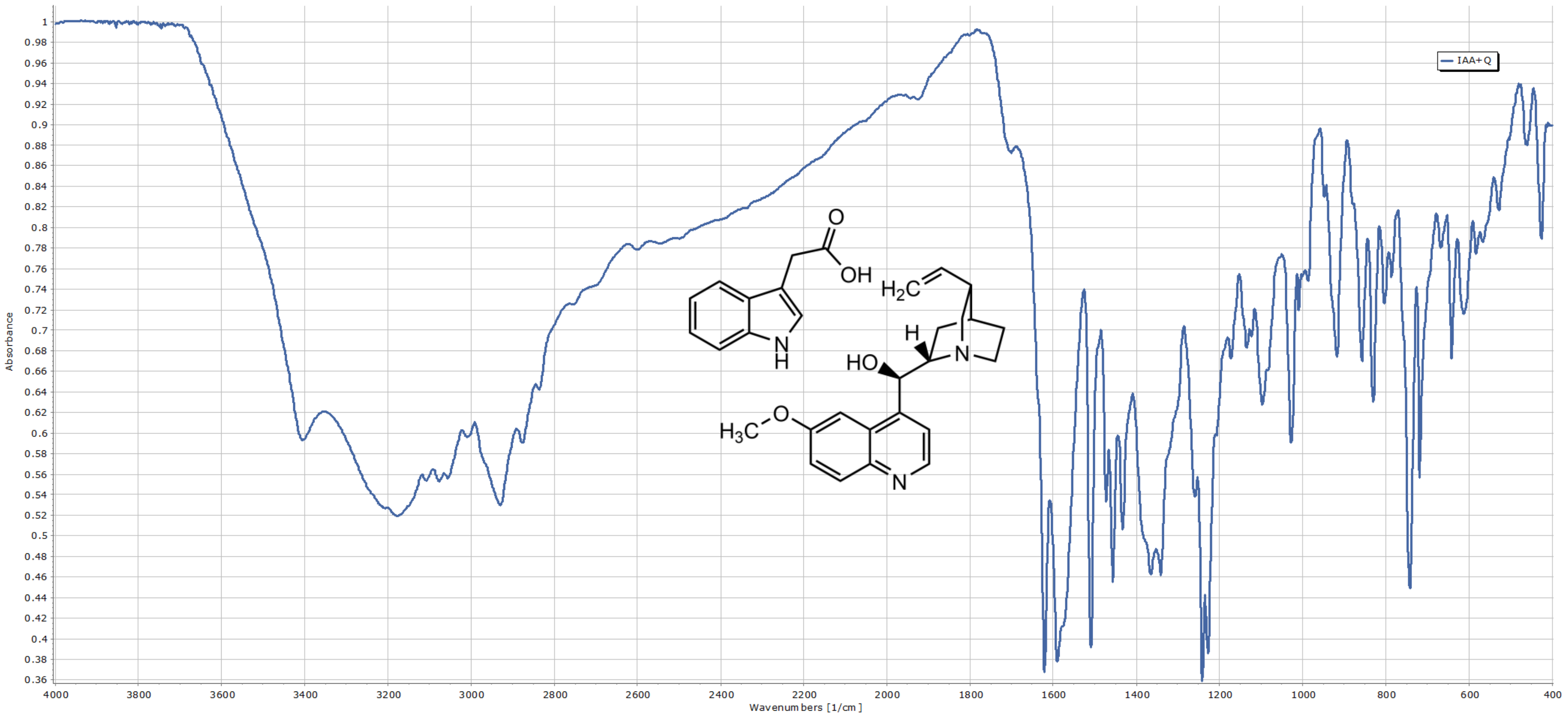

IR (KBr disc) ν [cm^{-1}] = 3405, 3178, 3108, 3076, 3056, 3007, 2927, 2877, 2836, 2757, 2600, 1621, 1590, 1509, 1473, 1457, 1433, 1365, 1341, 1241, 1228, 1173, 1134, 1097, 1027, 986, 918, 857, 830, 803, 786, 743, 718, 667, 642, 611, 582, 567, 527, 459, 425.

Figure S33. FT-IR spectrum of binary mixture of quinine and indole-3-acetic acid (IAA+Q)

2. COLLECTED DATA

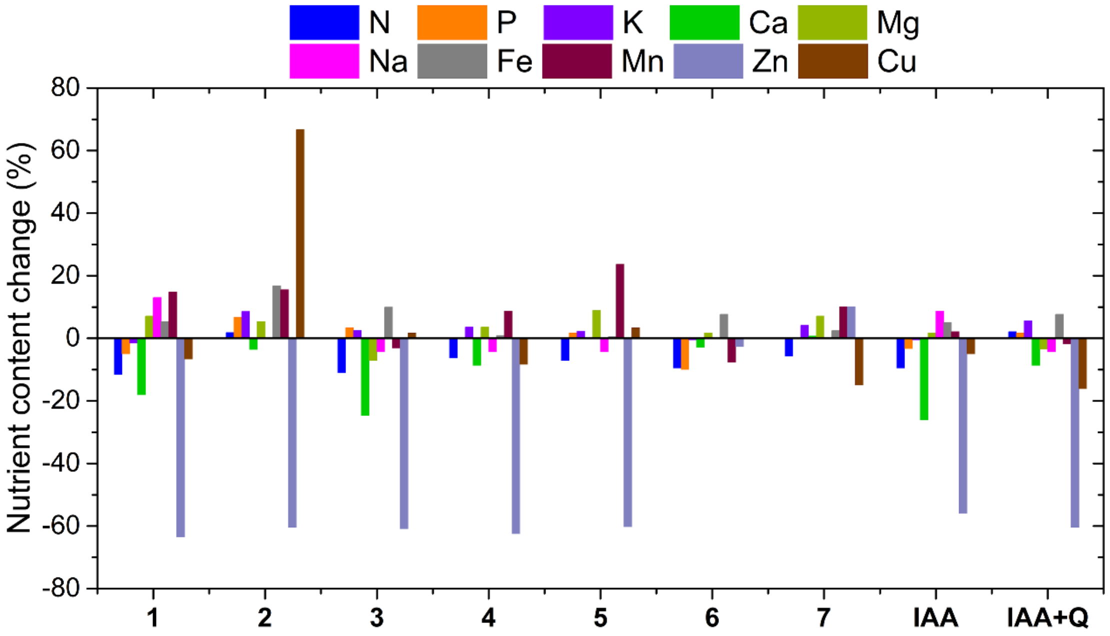

Figure S34. Changes in the content of all micronutrients and macronutrients in dry mass of lettuce plants grown with medium containing ILs **1**–**7** or reference substances (IAA, IAA+Q) compared to control plants

Table S1. Influence of the obtained ILs **1**–**7** and IAA and IAA+Q (reference substances) on the content of macronutrients and sodium in leaf dry matter

Compound	Content of macronutrients [g kg^{-1}]					
	N	P	K	Ca	Mg	Na
**1**	33.4 a	5.7 ab	55.0 a	11.3 ab	6.0 a	2.6 b
**2**	38.5 b	6.4 b	60.7 b	13.3 bc	5.9 a	2.3 ab
**3**	33.6 a	6.2 ab	57.3 ab	10.4 a	5.2 a	2.2 a
**4**	35.4 ab	6.0 ab	57.9 ab	12.6 bc	5.8 a	2.2 a
**5**	35.1 ab	6.1 ab	57.2 ab	13.8 c	6.1 a	2.2 a
**6**	34.2 ab	5.4 a	55.6 a	13.4 bc	5.7 a	2.3 ab
**7**	35.6 ab	6.0 ab	58.2 ab	13.9 c	6.0 a	2.3 ab
IAA	34.2 a	5.8 ab	55.6 a	10.2 a	5.7 a	2.5 ab
IAA+Q	38.6 b	6.1 ab	59.0 ab	12.6 bc	5.4 a	2.2 a
none (control)	37.8 ab	6.0 ab	55.9 ab	13.8 c	5.6 a	2.3 ab

a, b – values within columns marked with the same letter do not differ statistically

Table S2. Influence of the obtained ILs **1**–**7** and IAA and IAA+Q (reference substances) on the content of micronutrients in leaf dry matter

Compound	Content of micronutrients [mg kg^{-1}]			
	Fe	Mn	Zn	Cu
**1**	120.60 ab	92.20 b	29.27 a	5.35 a
**2**	133.73 b	92.83 b	31.77 a	10.00 b
**3**	125.87 ab	77.80 a	31.43 a	6.10 a
**4**	115.57 a	87.30 ab	30.13 a	5.47 a
**5**	115.05 a	99.27 b	31.85 a	6.15 a
**6**	123.33 ab	74.10 a	78.13 b	5.97 a
**7**	117.27 a	88.43 ab	88.43 c	5.10 a
IAA	120.35 ab	82.00 a	35.37 a	5.73 a
IAA+Q	123.30 ab	78.83 a	31.83 a	5.03 a
none (control)	114.53 a	80.27 a	80.27 bc	5.97 a

a, b – values within columns marked with the same letter do not differ statistically

Table S3. Influence of the obtained ILs **1**–**7** IAA and IAA+Q (reference substances) on the yielding of plants, dry matter (% DM) and relative water content (RWC) in leaves

Compound	Yielding [g plant^{-1}]	%DM	RWC
**1**	153.3 b	4.73 a	81.4 a
**2**	137.6 a	4.93 a	73.0 a
**3**	139.2 ab	4.72 a	72.7 a
**4**	138.6 ab	4.61 a	79.1 a
**5**	142.1 ab	4.68 a	74.7 a
**6**	136.1 a	4.82 a	75.4 a
**7**	137.0 a	4.80 a	77.2 a
IAA	152.1 b	4.82 a	80.9 a
IAA+Q	137.9 a	4.58 a	73.3 a
none (control)	149.3 ab	4.80 a	79.7 a

a, b – values within columns marked with the same letter do not differ statistically

Table S4. Effect of the ILs **1**–**7**, IAA and IAA+Q on selected indicators of chlorophyll fluorescence

Compound	F_0	F_M	F_V	F_V/F_0	F_V/F_M	*qP*
**1**	11 143.0 a	67241.0 a	56098.0 a	5.05 a	0.83 a	0.14 a
**2**	12 061.8 ab	76937.3 ab	64875.5 ab	5.38 a	0.84 a	0.14 a
**3**	10 062.5 a	59950.3 a	49887.8 a	4.95 a	0.83 a	0.26 a
**4**	11 696.0 ab	69906.8 ab	58210.8 ab	4.96 a	0.83 a	0.17 a
**5**	14 719.3 ab	92364.0 b	77644.8 b	5.29 a	0.84 a	0.28 a
**6**	12 086.0 ab	67883.0 a	55797.0 a	4.61 a	0.82 a	0.12 a
**7**	12 760.8ab	71540.5 ab	58779.8 ab	4.66 a	0.82 a	0.35 a
IAA	10 599.0 a	65461.0 a	54862.0 a	5.21 a	0.84 a	0.10 a
IAA+Q	11 663.5 ab	68517.0 a	56853.5 a	4.88 a	0.83 a	0.33 a
none (control)	16 117.5 b	91485.8 b	75368.3 b	4.84 a	0.83 a	0.19 a

a, b – values within columns marked with the same letter do not differ statistically

Table S5. Effect of the ILs **1**–**7**, IAA and IAA+Q on selected indicators of chlorophyll fluorescence

Compound	PI_{ABS}	*(1-Vj)/Vj*	*ABS/RC*	TR_O/RC	ET_O/RC	DI_O/RC	*Area*
**1**	2.98 a	1.28 ab	2.18 ab	1.82 ab	1.02 ab	0.36 a	25777309 ab
**2**	3.55 a	1.55 b	2.36 ab	1.99 ab	1.21 ab	0.37 a	27547401 ab
**3**	3.86 a	1.52 b	1.96 a	1.63 a	0.98 a	0.33 a	22412195 a
**4**	3.38 a	1.27 b	2.24 ab	1.87 ab	1.12 ab	0.38 a	28442944 ab
**5**	2.84 a	1.37 ab	2.62 ab	2.20 ab	1.27 b	0.42 a	34133986 ab
**6**	2.32 a	1.07 a	2.15 ab	1.77 ab	0.91 a	0.38 a	34817649 b
**7**	2.54 a	1.32 ab	2.51 ab	2.06 ab	1.16 ab	0.45 a	31780344 ab
IAA	3.36 a	1.35 ab	2.19 ab	1.83 ab	1.04 ab	0.36 a	24093867 ab
IAA+Q	2.77 a	1.39 ab	2.72 ab	2.25 ab	1.30 b	0.47 a	24454585 ab
none (control)	2.77 a	1.42 b	2.80 b	2.31 b	1.34 b	0.50 a	33599867 ab

a, b – values within columns marked with the same letter do not differ statistically
